# Genomic Adaptations to Salinity Resist Gene Flow in the Evolution of Floridian Watersnakes

**DOI:** 10.1093/molbev/msaa266

**Published:** 2020-10-09

**Authors:** Rhett M Rautsaw, Tristan D Schramer, Rachel Acuña, Lindsay N Arick, Mark DiMeo, Kathryn P Mercier, Michael Schrum, Andrew J Mason, Mark J Margres, Jason L Strickland, Christopher L Parkinson

**Affiliations:** 1 Department of Biological Sciences, Clemson University, Clemson, SC; 2 Department of Biology, University of Central Florida, Orlando, FL; 3 Department of Biology, City College of New York, New York, NY; 4 PhD Program in Biology, The Graduate Center of the City University of New York, New York, NY; 5 Department of Organismic and Evolutionary Biology, Harvard University, Cambridge, MA; 6 Department of Integrative Biology, University of South Florida, Tampa, FL; 7 Department of Biology, University of South Alabama, Mobile, AL; 8 Department of Forestry and Environmental Conservation, Clemson University, Clemson, SC

**Keywords:** ecological niche, introgression, local adaptation, selection, speciation

## Abstract

The migration-selection balance often governs the evolution of lineages, and speciation with gene flow is now considered common across the tree of life. Ecological speciation is a process that can facilitate divergence despite gene flow due to strong selective pressures caused by ecological differences; however, the exact traits under selection are often unknown. The transition from freshwater to saltwater habitats provides strong selection targeting traits with osmoregulatory function. Several lineages of North American watersnakes (*Nerodia* spp.) are known to occur in saltwater habitat and represent a useful system for studying speciation by providing an opportunity to investigate gene flow and evaluate how species boundaries are maintained or degraded. We use double digest restriction-site associated DNA sequencing to characterize the migration-selection balance and test for evidence of ecological divergence within the *Nerodia fasciata*-*clarkii* complex in Florida. We find evidence of high intraspecific gene flow with a pattern of isolation-by-distance underlying subspecific lineages. However, we identify genetic structure indicative of reduced gene flow between inland and coastal lineages suggesting divergence due to isolation-by-environment. This pattern is consistent with observed environmental differences where the amount of admixture decreases with increased salinity. Furthermore, we identify significantly enriched terms related to osmoregulatory function among a set of candidate loci, including several genes that have been previously implicated in adaptation to salinity stress. Collectively, our results demonstrate that ecological differences, likely driven by salinity, cause strong divergent selection which promotes divergence in the *N. fasciata-clarkii* complex despite significant gene flow.

## Introduction

Once thought to be rare, speciation with gene flow is now considered exceedingly common across the tree of life (Nosil 2008; Barth et al. 2020). Speciation genomics has uncovered evidence of rampant introgression among species and reticulate evolution of lineages, lending insights into how speciation progresses (Feder et al. 2012; Campbell et al. 2018; Mason et al. 2019). Although gene flow is typically considered a homogenizing force, reducing divergence among species and impeding local adaptation (Endler 1977; Slatkin 1987; Garcia-Ramos and Kirkpatrick 1997; Lenormand 2002), many cases exist in which gene flow promotes divergence by introducing genetic variation, or speciation proceeds in spite of gene flow (Slatkin 1987; Ingvarsson 2001; Garant et al. 2007; Lamichhaney et al. 2015; Pease et al. 2016; Pfeifer et al. 2018; Dean et al. 2019; Margres et al. 2019; Choi et al. 2020). Identifying the processes which underly speciation with gene flow is a key goal in evolutionary biology, but the interaction of evolutionary forces can be complex and vary dramatically across systems (Garant et al. 2007).

Strong selection caused by ecological differences between populations can lead to local adaptation and promote speciation (Schluter 2000; Rundle and Nosil 2005). If selection is weak, gene flow may result in genetic swamping whereby locally fit genotypes are driven to extinction. Alternatively, strong selection may overcome the homogenizing force of gene flow and maintain diversity or facilitate divergence. Therefore, migration and selection sit in a balance which governs the evolution of a given lineage (Haldane 1930; Wright 1931; Yeaman and Whitlock 2011; Savolainen et al. 2013; Tigano and Friesen 2016). Ecological speciation provides a set of predictions which can be explicitly tested with regards to gene flow and selection. Under ecological speciation, the effect of isolation-by-environment (IBE) should be greater than isolation-by-distance (IBD) since species are able to persist despite close proximity (Wang and Bradburd 2014). Detecting signatures of ecological speciation requires characterizing the migration-selection balance and ecological divergence in order to determine the prevalence of gene flow, characterize the ecological factors influencing divergence, and identify loci under selection that are resistant to the homogenizing effects of gene flow. Identifying historical or ongoing ecological speciation can therefore be more readily accomplished in systems where environmental conditions promote differential selection pressures on putatively divergent lineages.

The transition from freshwater to saltwater can provide strong divergent selection due to the physiologically harsh, high-saline environment (Hong et al. 2019). For example, less than 1% of extant reptiles extensively utilize saltwater habitats (Rasmussen et al. 2011; Peng et al. 2020). In lineages that occur in saltwater, selection has targeted genes related to osmoregulatory function and ion transport (Zhang et al. 2017; Hong et al. 2019; Mansouri et al. 2019; Lee et al. 2020); therefore, lineages that occur in freshwater and saline environments provide an opportunity to study ecological speciation. At least four North American watersnake (*Nerodia* spp.) lineages are known to occur in high-salinity environments (Rasmussen et al. 2011). Watersnakes are also widespread, abundant, and many lineages are sympatric—providing ample opportunity for gene flow, including inferred hybridization, and enabling the investigation of the formation, maintenance, and/or degradation of species boundaries (Conant 1963; Lawson et al. 1991; Mebert 2008). The *Nerodia fasciata-clarkii* complex, in particular, is differentiated by only a few morphological characters and presumed ecological differences, with *N. fasciata* (Southern Watersnake) predominantly occupying—but not limited to—freshwater habitats and *N. clarkii* (Salt Marsh Snake) occupying coastal, mostly saltwater habitats (Lawson et al. 1991; see Gibbons and Dorcas [2004] for full distributions of each taxon; USFWS [2008]). Moreover, unlike most marine reptiles, *N. clarkii* does not have salt glands and display no clear physiological adaptations to high-salinity environments (Pettus 1958, 1963; Dunson 1980; Babonis et al. 2011). Despite this, *N. clarkii* is able to maintain its plasma ion balance more effectively than its sister species; therefore, the mechanisms that allow *N. clarkii* to maintain osmotic or ionic balance remain elusive (Babonis et al. 2011). Environmental and behavioral differences between *N. fasciata* and *N. clarkii* suggest local adaptation, but few studies have tested whether genetic or molecular differences occur between the two species (Lawson et al. 1991; Jansen 2001) and no studies have tested for an association between genetic variation and salinity tolerance.

**Fig. 1.**
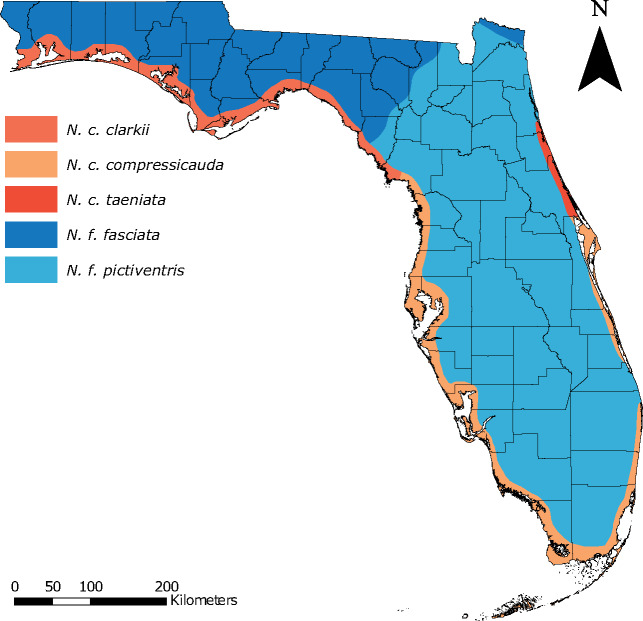
Distribution map for the five subspecies within the *Nerodia fasciata-clarkii* complex in Florida. Colors are maintained throughout the manuscript.

Characterizing the migration-selection balance of the *N. fasciata-clarkii* complex also has critical implications for conservation biology. Within Florida, *N. fasciata* and *N. clarkii* are composed of five subspecies (*N. f. fasciata*, *N. f. pictiventris*, *N. c. clarkii*, *N. c. compressicauda*, and *N. c. taeniata*; fig. 1), with *N. c. taeniata* listed as a federally threatened subspecies (USFWS 2008). Previous research has questioned the separation of *N. fasciata* and *N. clarkii* because of low interspecific divergence and substantial morphological intergradation, hypothesized to be the result of high gene flow (Carr and Goin 1942; Lawson et al. 1991; USFWS 2008; reviewed in Territo 2013). The lack of clear morphological differentiation and hypothesized gene flow among lineages has made delineating conservation units and evaluating the recovery status of *N. c. taeniata* difficult (USFWS 2008), necessitating the use of genomics and current approaches to inform future decision-making.

Given the ecologically divergent nature of the *N. fasciata*-*clarkii* complex, we hypothesize that adaptations to saline environments are able to resist the homogenizing effects of gene flow between *N. fasciata* and *N. clarkii* (Lawson et al. 1991). If adaptation facilitates differentiation in spite of gene flow, we expect to find genetic structure corresponding to environmental differences (i.e., salinity) between *N. fasciata* and *N. clarkii.* Specifically, we expect the influence of IBE to outweigh patterns of IBD between species, but not between subspecies/populations within a species. Additionally, we expect to find evidence of ecological divergence among lineages and genomic signatures of selection associated with loci related to osmoregulatory functions. Therefore, we aim to 1) identify population structure and infer the evolutionary history of the group, 2) characterize the role of gene flow and hybridization in shaping these patterns, 3) test for ecological niche divergence and determine if genetic differentiation or admixture is related to niche differentiation, 4) identify loci under selection and determine if they are related to osmoregulatory function, and 5) evaluate the implications of our findings for conservation in light of the threatened status of *N. c. taeniata*. To do this, we use a combination of Sanger sequencing and double digest restriction-site associated DNA sequencing (ddRADseq) to infer population structure and phylogenetic history. We use the ddRADseq data and multiple analytical methods to provide evidence of gene flow, including hybrid lineage formation, and search for outlier loci associated with osmoregulatory function. We use publicly available environmental data, including soil cation exchange capacity as a proxy for salinity, to test for differences in ecological niche and ask whether salinity may be a driver for genetic differentiation or admixture between the species. Finally, we utilize several morphological characters to explore their utility in diagnosing species or subspecies within this complex. For ease, we utilize the existing nomenclature to communicate and discuss our results.

## Results

### Population Structure

**Fig. 2.**
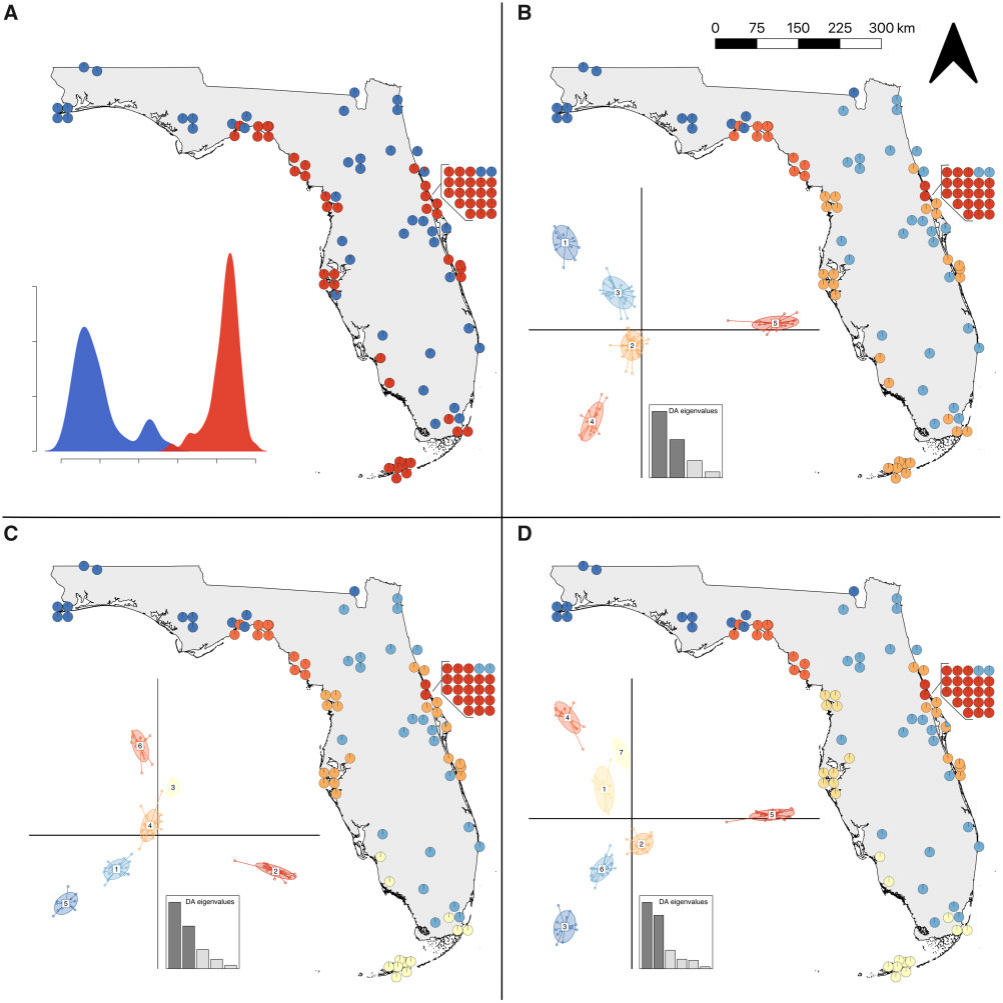
*DAPC* population structure for 118 snakes across Florida. (*A*) *K* = 2 shows that genetic clustering is similar to the species distributions (see fig. 1). Population structure at (*B*) *K* = 5 shows that genetic clustering is similar to subspecies distributions (see fig. 1). The best supported *DAPC* model (*C*) *K* = 6 separates the *N. c. compressicauda* population into central and southern groups. (*D*) At *K* = 7, the eastern and western *N. c. compressicauda* clades separate. Inset density and scatter plots represent separation along discriminant axes and the DA eigenvalues describe how much discriminating ability the model possesses. Not all 118 points may be visible in maps since some points overlap.

To assess population structure, we performed ddRADseq on 131 individuals including one outgroup (*Thamnophis saurita*). Our final filtered data set consisted of 119 individuals (including the outgroup) and 7,057 single nucleotide polymorphisms (SNPs). Excluding the outgroup, we identified population clusters using a discriminant analysis of principal components (*DAPC*; Jombart et al. 2010). K-means clustering identified *K* = 6 as the best-fit model with *K* = 5 and *K* = 7 performing similarly (ΔBIC <2; supplementary fig. S1, Supplementary Material online). We used all three models of population structure (i.e., *K* = 5, 6, and 7) for downstream analyses. In addition, we examined the geographic structure at *K* = 2 to assess if genetic clustering accurately represents the distribution of the two species (fig. 2*A*). At *K* = 2, clustering followed a coastal/inland dichotomy similar to the approximate ranges of *N. clarkii* and *N. fasciata* (figs. 1 and 2A). Similarly, at *K* = 5, the population clustering approximates the ranges of the five subspecies in Florida (figs. 1 and 2*B*). In addition to general concordance between the *DAPC* clusters and expected distributions of the species/subspecies (figs. 1 and 2), we also found that species assignment at capture had 92% concordance with *K* = 2 *DAPC* clusters and subspecies assignment had 88% concordance with *K* = 5 *DAPC* clusters (ignoring the seven individuals included in ddRADseq sampling with uncertain taxonomic assignment; supplementary table S1, Supplementary Material online). However, it should be noted that assignment at capture was not performed blind with respect to sampling location. Accordingly, we utilize the current taxonomy to refer to concordant genetic clusters to communicate the remainder of our results. At *K* = 6 and *K* = 7, only *N. c. compressicauda* was impacted (fig. 2*C* and *D*). At *K* = 6, the best-fit model, the southern portion of the range was separated from central Florida populations, followed by separation of the east and west coasts at *K* = 7 (fig. 2*C* and D).

**Fig. 3.**
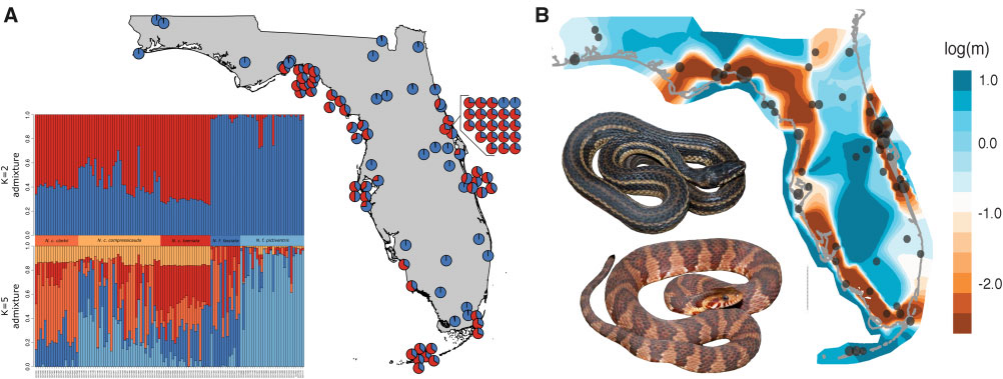
(A) *conStruct* population structure and admixture at *K* = 2 with additional *K* = 5 admixture plot showing little structure at *K* = 5. (*B*) *EEMS* for the *N. fasciata-clarkii* complex. High migration is represented by shades of blue; low migration is represented by red–orange shades. *EEMS* demonstrates increased gene flow within and decreased gene flow between the coastal and inland populations. Photos of *N. c. clarkii* and *N. f. fasciata* by K. Wray.

To dissociate population structure from continuous clines of genetic variation, we ran *conStruct* (Bradburd et al. 2018). Cross-validation analysis in *conStruct* found that spatially aware analyses outperformed nonspatial analyses, suggesting that some divergence in the *N. fasciata-clarkii* complex can be attributed to IBD (supplementary fig. S2, Supplementary Material online). We examined the contribution of each additional layer (*K*) and found that the majority of the data are explained with two layers, suggesting that there are two significantly different IBD clines in our data (*K* = 2; supplementary fig. S2, Supplementary Material online). Geographic structure of these data followed the coastal-inland dichotomy previously identified by *DAPC* (*K* = 2) and was similar to the species ranges of *N. clarkii* and *N. fasciata* (figs. 1 and 3A). *conStruct* also identified high admixture proportion between the two species, with *N. clarkii* frequently having upwards of 50% admixture from *N. fasciata* (fig. 3*A*); however, there was little evidence of admixture within *N. fasciata* from *N. clarkii* (fig. 3*A*). The high admixture proportion detected in *N. clarkii* but not *N. fasciata* could be due to unidirectional gene flow, shared ancestral polymorphism, or differences in effective population size (Lawson et al. 2018). Nonetheless, we identified substantial allelic variation unique to *N. clarkii*, which highlights the shared ancestry of coastal lineages as well as their genetic distinction from *N. fasciata.*

### Phylogenetics

Initial phylogenetic analysis of the mitochondrial gene, *cytb*, for 268 individuals and 20 outgroups found no support for subspecies or species monophyly (supplementary fig. S3, Supplementary Material online). Further analysis with two additional mitochondrial genes (*ND1* and *ND4*), two nuclear genes (*PRLR* and *TATA*), and two anonymous loci (*M* and *E*) for 85 individuals and 11 outgroups confirmed these results. Overall, single-gene sequencing provided little resolution within the *N. fasciata-clarkii* complex with large polytomies, low support, and the only clear distinction occurring between continental and peninsular populations (supplementary fig. S3, Supplementary Material online) as is commonly seen in taxa with similar distributions (Soltis et al. 2006; Strickland et al. 2014; Margres et al. 2019).

**Fig. 4.**
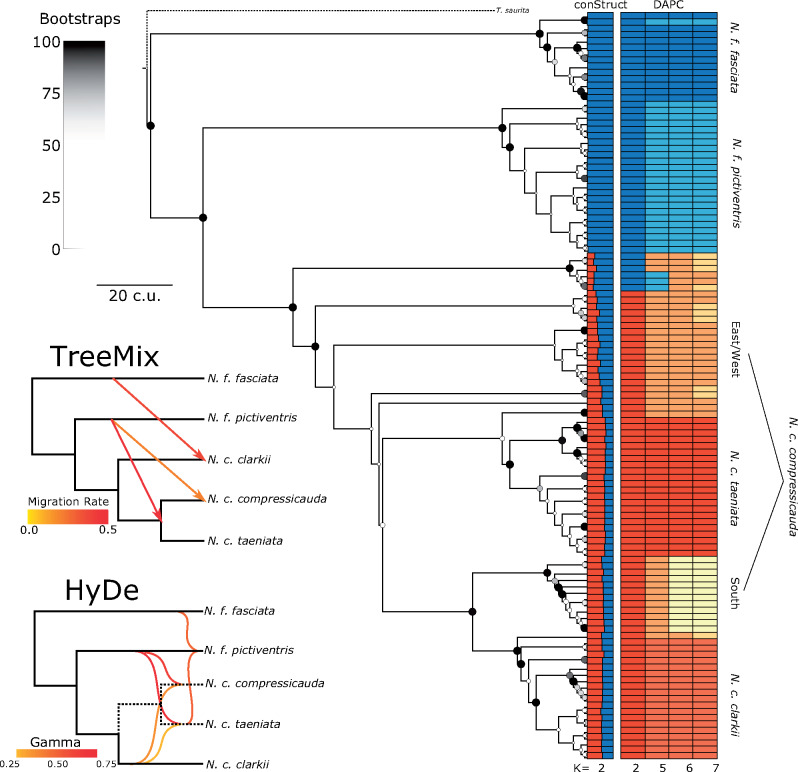
*SVDquartets* inferred phylogeny with *conStruct* and *DAPC* results annotated. Nodal support is represented by color and size with large, black dots representing 100% bootstrap support and small, white points representing 0%. See supplementary figure S7, Supplementary Material online, for a cladogram with numerical support values. *SVDquartets* and *Treemix* species tree are also shown for *K* = 5 with three migration edges leading from *N. fasciata* to *N. clarkii*, particularly from *N. f. pictiventris* to *N. c. compressicauda*. *HyDe* similarly detected three significant hybridization events, position of hybridization edges on branches is arbitrary.

To assess the phylogenomic history of the group, we generated a biallelic SNP alignment and used *SVDquartets* (Chifman and Kubatko 2014). The resulting phylogeny demonstrated strong support for several monophyletic clades generally corresponding to the *DAPC* clusters (fig. 4). Primarily, a lineage of individuals, all but one of which were identified as *N. f. fasciata* at capture and by *DAPC*, was found to be sister to the remaining taxa. Following this, a branching event led to a lineage corresponding to *N. f. pictiventris*, rendering *N. fasciata* paraphyletic (fig. 4). Sister to *N. f. pictiventris* is a large clade of individuals with high admixture proportion as inferred by *conStruct* (fig. 4). Six individuals with ambiguous *DAPC* assignments and with 63–74% *conStruct* admixture proportions from *N. fasciata* form the sister clade to individuals consistently assigned to *N. clarkii*. Within the *N. clarkii* clade, several branching events occur associated with the central *N. c. compressicauda DAPC* cluster, rendering this cluster paraphyletic (fig. 4). These small, paraphyletic clades generally have low support (<50%) and could be the result of a hard polytomy or admixture from *N. fasciata* (fig. 4). Sister to the paraphyletic clades corresponding to central *N. c. compressicauda* is a moderately supported split leading to a clade consisting of *N. c. taeniata*, *N. c. clarkii*, and southern *N. c. compressicauda* (fig. 4). However, three individuals assigned to central *N. c. compressicauda* were also found sister to either the *N. c. taeniata* or *N. c. clarkii* clades, further demonstrating their uncertain phylogenetic placement and paraphyly of *N. c. compressicauda*.

In addition, we used the *K* = 5, 6, and 7 *DAPC* clusters to construct species trees with *SVDquartets*. All three species trees had 100% bootstrap support for all nodes. The *K* = 5 tree similarly identified *N. f. fasciata* as sister to the remaining taxa followed by a branching event leading to *N. f. pictiventris* and *N. clarkii* (fig. 4). Within *N. clarkii*, *N. c. clarkii* was found to be sister to a branching event of *N. c. compressicauda* and *N. c. taeniata* (fig. 4). At *K* = 6 and *K* = 7, central *N. c. compressicauda* was found sister to the remaining *N. clarkii* clade (supplementary fig. S4, Supplementary Material online).

### Gene Flow

To test for migration events in the evolutionary history of the Florida *N. fasciata-clarkii* complex, we ran *Treemix* (Pickrell and Pritchard 2012) on the *DAPC* cluster assignments at *K* = 5, 6, and 7. The best network at *K* = 5 matched the species tree inferred by *SVDquartets*, but contained three migration edges suggesting gene flow from *N. fasciata* into *N. clarkii* (fig. 4). One migration edge led from *N. f. fasciata* to *N. c. clarkii*, one migration edge was inferred from *N. f. pictiventris* to the base of *N. c. taeniata* and *N. c. compressicauda*, and one migration edge was inferred from *N. f. pictiventris* directly to *N. c. compressicauda* (fig. 4). At *K* = 6, the best network did not match the *SVDquartets* species tree, but also contained three migration edges; one leading from *N. f. pictiventris* to *N. f. fasciata*, one leading from *N. f. pictiventris* to *N. c. compressicauda*, and one leading from the base of the tree to *N. c. clarkii* (supplementary fig. S4, Supplementary Material online). At *K* = 7, the best network contained five migration edges, demonstrating the tremendous amount of gene flow occurring within this complex (supplementary fig. S4, Supplementary Material online).

To further identify gene flow and detect potential hybrid origin of populations or subspecies, we used *HyDe* (Blischak et al. 2018) with all possible triples (i.e., parent/hybrid combinations) based on the clusters identified by *DAPC*. We detected evidence of hybrid origin for *N. f. pictiventris*, *N. c. compressicauda*, and *N. c. taeniata* populations with gamma (ɣ, probability of assignment to one parent) ranging from 0.44 to 0.75 (fig. 4 andsupplementary figs. S4 and S5, Supplementary Material online). However, populations identified as hybrids also functioned as parents for other populations (fig. 4 and supplementary figs. S4 and S5, Supplementary Material online); therefore, the evolutionary history leading to these populations appears to be a result of rampant hybridization among nearly all populations. Interestingly, *N. c. taeniata* was consistently found to be the result of hybridization between *N. c. clarkii* and *N. f. pictiventris* which may support a previous hypothesis suggesting that *N. c. taeniata* intergrades with neighboring populations and is derived from *N. c. clarkii*, given color pattern similarities (fig. 4 and supplementary figs. S4 and S5, Supplementary Material online; Carr and Goin 1942).

To visualize the patterns of gene flow across Florida, we used *EEMS* to estimate an effective migration surface ( Petkova et al. 2016). *EEMS* clearly demonstrates a strong reduction in gene flow between coastal and inland environments and additional weaker reductions along subspecies geographic boundaries (fig. 3*B*). Specifically, *EEMS* identifies a nearly panmictic inland population, suggesting high gene flow between subspecies of *N. fasciata* (fig. 3*B*). However, a slight reduction in gene flow can be seen near the Suwanee Straight, consistent with the identification of mainland and peninsular clades in the phylogenetic analyses (fig. 3*B* and supplementary fig. S3, Supplementary Material online). Similarly, *EEMS* identifies high gene flow among coastal populations and subspecies of *N. clarkii*. A slight barrier may exist between *N. c. clarkii* in northwestern Florida and *N. c. compressicauda*. There is an additional break for *N. clarkii* along the southeast coast of Florida separating eastern and southern clades of *N. c. compressicauda* (fig. 3*B*). Previously, *N. c. compressicauda* was thought to be absent from southeast Florida coastlines and, due to heavy urbanization, there is little habitat remaining. However, this break may be due to incomplete sampling since *N. c. compressicauda* was recently discovered in this area (Holbrook et al. 2016). Finally, *EEMS* identifies areas of high introgression between *N. fasciata* and *N. clarkii* along both the east and west-central coasts (fig. 3*B*).

### Ecological Niche Divergence

To assess ecological niche divergence between *N. fasciata* and *N. clarkii*, we first used *Humboldt* (Brown and Carnaval 2019) to perform Niche Overlap Tests (NOT) and Niche Divergence Tests (NDT). We downloaded 7,320 occurrence records for *N. fasciata* and *N. clarkii* from the Global Biodiversity Information Facility (1,216 *N. clarkii* and 6,104 *N. fasciata*; GBIF.org 2019) and removed records outside of Florida and each species distribution resulting in a final data set of 3,623 records (649 *N. clarkii* and 2,974 *N. fasciata*). The results of *Humboldt* were inconclusive and prone to Type I error due to niche truncation or observations being located close to the edge of the available environmental space, leading to the inability to obtain appropriate background data (supplementary fig. S6, Supplementary Material online; Brown and Carnaval 2019).

**Fig. 5.**
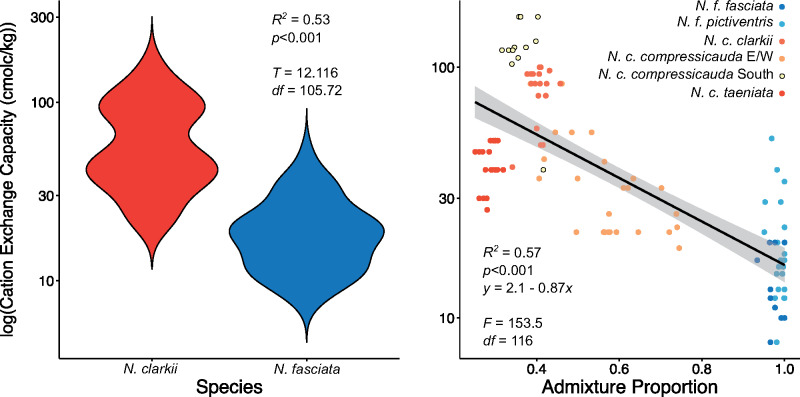
Violin plot demonstrating the differences in cation exchange capacity of the soil—used as a metric of salinity—between *N. fasciata* and *N. clarkii* based on *DAPC* assignment at *K* = 2. Regression of *conStruct* admixture proportion at *K* = 2 (proportion assigned to *N. fasciata*) with cation exchange capacity of the soil. Points are colored by *DAPC* assignment at *K* = 6.

Therefore, we further explored the six most informative variables identified by *Humboldt*. These variables primarily included landcover variables such as the amount of regularly flooded vegetation and open water, which was greater in *N. clarkii* (supplementary fig. S6, Supplementary Material online). Notably, however, we found that cation exchange capacity of the soil—a variable highly correlated with salinity (Saidi 2012)—was much higher for *N. clarkii* (supplementary fig. S6, Supplementary Material online). To determine if this pattern was maintained in our genetic clusters, we extracted cation exchange capacity data for all 118 genetic samples and found significant differences between the two species (*DAPC K* = 2; *R*^2^ = 0.53; *P *<* *0.001; fig. 5). Furthermore, we found a significant relationship with the *conStruct* admixture proportion suggesting that admixture may be limited by salinity (*R*^2^ = 0.57; *P *<* *0.001; fig. 5). However, admixture proportions might represent shared ancestry rather than gene flow. Therefore, to disentangle the influence of IBD and IBE caused by salinity on genetic dissimilarity, we performed a series of Mantel and partial Mantel tests using all individuals and separately for each species (*DAPC K* = 2). If IBE plays a role in species divergence, we expect a significant correlation when comparing all individuals, but not within each species. Mantel tests confirm that IBD strongly influences our data both across and within a single species (0.38 ≤ *r *≤* *0.65; *P *=* *0.001). Partial Mantel tests confirm that genetic dissimilarity is associated with differences in salinity when looking across species (*r *=* *0.15; *P *=* *0.001), but not within *N. fasciata* (*r* = −0.30; *P *=* *1) or *N. clarkii* (*r *=* *0.03; *P *=* *0.25).

### Scans for Selection Signatures

**Fig. 6.**
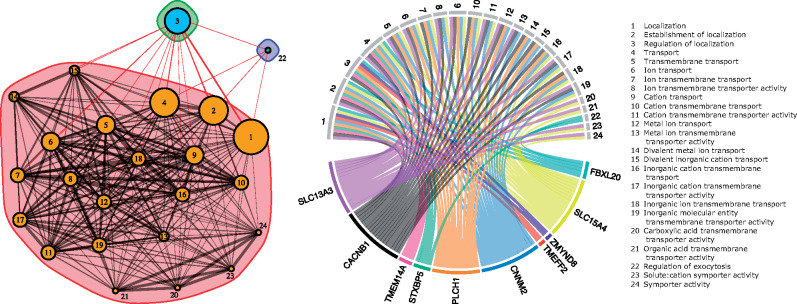
Left: Network generated by *ShinyGO* of all significantly enriched GO terms for genes shared across *BayeScan* scans. Two nodes are connected if they share greater than 20% of their genes. Node size corresponds to size of the gene sets and edge thickness corresponds to the number of overlapped genes. The colored envelopes represent modules with dense nodal connections, but sparse connections to other modules. Right: Genes associated with ion, cation, and transmembrane transport which are likely related to osmoregulatory adaptations associated with coping with saline environments in *N. clarkii*.

Given the ecological differences observed between *N. fasciata* and *N. clarkii*, we sought to identify genes potentially contributing to their divergence. We used *BayeScan* with a reduced data set, only including *N. clarkii* with <50% admixture proportion from *N. fasciata*. *BayeScan* identified 62 outlier SNPs between *N. fasciata* (*n *=* *41) and *N. clarkii* (*n *=* *60) which corresponded to 41 loci (*P *<* *0.05; supplementary table S3, Supplementary Material online). After searching for genes within 2 kb of BLAST matches in the *Thamnophis elegans* reference genome (GCF_009769535.1), we identified 31 potential genes of interest (supplementary table S4, Supplementary Material online). Gene ontology (GO)-enrichment analysis identified a network of 24 significantly enriched GO terms (FDR <0.05) corresponding to ten genes (fig. 6). The majority of enriched terms are related to transmembrane transport of ions and metals. Specifically, these terms included cation transmembrane transport (GO:0098655), cation transport (GO:0006812), metal ion transport (GO:0030001), and transmembrane transport (GO:0055085; fig. 6). The ten associated genes likely correspond to changes in osmoregulatory abilities between the two species and the ability to cope with high-salinity environments (fig. 6).

### Morphology

To evaluate our findings in light of current taxonomy and morphological variation, we measured 11 meristic and dimensional features for 137 specimens across the range (fig. supplementary S8*A*, Supplementary Material online) and performed principal component analysis (PCA). PC1 accounted for 22.7% of the variation and was most influenced by the number of postocular scales; PC2 accounted for 16.4% of the variation and was most influenced by the number of subcaudal scales. Model-based clustering in the R package *mclust* (Scrucca et al. 2016) identified two morphological clusters; however, these clusters overlapped heavily in PC space (supplementary fig. S8*B*, Supplementary Material online). To assess if species or subspecies formed morphological clusters we plotted these variables in PC space (supplementary fig. S8*C* and *D*, Supplementary Material online) and performed AIC linear model comparison on the first two principal components using the species assignment, subspecies assignment, sex, age, and capture latitude as explanatory variables. We found that species assignment at capture was the best predictor of PC1 (supplementary fig. S9*B*, Supplementary Material online), but sex was the best predictor of PC2 (supplementary fig. S9*C*, Supplementary Material online). When only using morphological data with corresponding genetic data (*n *=* *21), we found that *conStruct* admixture proportions (*K* = 2) was the best predictor of PC1, demonstrating general agreement between the morphological and genetic differentiation of *N. fasciata* and *N. clarkii*, with genetically intermediate individuals also having intermediate phenotypes (supplementary fig. S9*A*, Supplementary Material online). Additionally, using a Mantel test, we found a significant correlation between genetic dissimilarity and morphological distance (*r *=* *0.24; *P *=* *0.01). However, the amount of morphological difference between species was small and even less between subspecies. The main difference between species is related to the number of postocular scales, with a difference of only one scale between the two species.

## Discussion

The formation and maintenance of lineages are governed by the homogenizing effects of gene flow and reinforcement of genomic divergence due to ecological and environmental selection (Rundle and Nosil 2005; Yeaman and Whitlock 2011; Tigano and Friesen 2016). We provide compelling evidence that pervasive gene flow and environmental selection, likely caused by differences in salinity, shape the evolutionary history of the *N. fasciata-clarkii* complex. Specifically, we find that IBD explains patterns of population structure, but IBE—caused by differences between inland and coastal habitats and subsequent selection on osmoregulatory genes—better explains broader genetic structure despite introgression from *N. fasciata* into *N. clarkii* and putative hybrid formation of some lineages.

Historic and contemporary gene flow play vital roles in mediating outcomes of incipient speciation, including the establishment of reproductive barriers or, conversely, lineage fusion (Nosil 2008; Tigano and Friesen 2016). Because previous studies have identified hybridization among lineages within the *N. fasciata-clarkii* complex (Conant 1963; Lawson et al. 1991), we expected to find evidence of gene flow among lineages. Our explicit tests of gene flow and population structure analyses find strong evidence of pervasive gene flow and admixture across the *N. fasciata-clarkii* complex. It is well-known that gene flow generates conflicts in phylogenetic signal (Degnan and Rosenberg 2009; Cui et al. 2013; Pease et al. 2016; Mason et al. 2019); therefore, gene flow is likely responsible for the limited nodal support recovered in our phylogenies and further demonstrates the most probable cause of historical phylogenetic discrepancies, with regard to the separation of *N. fasciata* and *N. clarkii*, and limitations of single-gene phylogenies. However, gene flow also played a critical role in the formation of several lineages, particularly *N. c. compressicauda* and *N. c. taeniata*, as demonstrated by *HyDe* (fig. 4). This suggests that gene flow among lineages might, in some cases, facilitate diversification possibly through the introduction of genetic variation. Instances where gene flow and hybridization promote genetic diversity and adaptation by introducing genetic variation are well-documented, including adaptation to high altitudes from Denisovans into humans in Tibet (Huerta-Sánchez et al. 2014; Tigano and Friesen 2016; Patton et al. 2020). Gene flow was widespread among *Nerodia* lineages, but it was asymmetrical. *conStruct* showed that a large proportion of *N. fasciata* alleles were shared with *N. clarkii*, but very few *N. clarkii* alleles were shared with *N. fasciata* (fig. 3). Although *conStruct* admixture proportions might instead be a result of shared ancestry or differences in effective population size (Lawson et al. 2018), *TreeMix* and *HyDe* analyses similarly indicated that gene flow primarily occurred from *N. fasciata* lineages to *N. clarkii* lineages (fig. 4). Unfortunately, determining the exact nature of the timing and extent of gene flow among these watersnakes requires expanded sampling to include lineages outside the range of Florida as well as explicit tests of alternate demographic histories. Nonetheless, the persistence of *N. clarkii*-specific alleles despite high gene flow suggests selective advantage of these genes.

We found evidence that differential selection based on ecological divergence contributed to the maintenance of genomic divergence between lineages. *Nerodia fasciata* and *N. clarkii* have long been distinguished by putative ecological differences, but we provide empirical evidence of niche differentiation. As hypothesized, cation exchange capacity in the soil—a proxy for salinity—was implicated as an environmental variable potentially limiting admixture and facilitating genetic differentiation of the two species through IBE (fig. 5). These findings are consistent with *EEMS* as the primary barriers to gene flow occur near inland-coastal ecotones where habitat type and salinity may rapidly shift (fig. 3). Salinity might provide a threshold under which admixture can occur, with high-salinity environments having little admixture. However, salinity and admixture are more likely to represent a continuous cline with temporal variation in salinity—which we did not measure—also playing a large role in the degree of admixture between the two species. Further support for the role of salinity driving divergence of these watersnakes was apparent in our scans for signatures of selection, in which the majority of the loci detected as outliers corresponded to genes associated with osmoregulatory function, consistent with previous experimental studies on adaptive responses to salinity stress in plants, fish, and turtles (Zhang et al. 2017; Hong et al. 2019; Mansouri et al. 2019; Lee et al. 2020). For example, we identified two genes from the solute-carrier (SLC) family which have been previously identified in responders to salinity-stress (Zhang et al. 2017; Hong et al. 2019). Unfortunately, establishing causal relationships between our identified candidate genes and increased salt tolerance will require experimental study. Similarly, it would be interesting to investigate the possibility of convergent or parallel evolution of salt tolerance in *N. clarkii* and other salt-tolerant reptiles. Nonetheless, strong divergent selection caused by ecological divergence in salinity has likely led to local adaptation and we hypothesize it contributes to divergence in the *N. fasciata-clarkii* complex.

Reconciling the pattern of asymmetric gene flow from *N. fasciata* into *N. clarkii* with the salinity-driven adaptations in *N. clarkii* is counterintuitive since *N. clarkii* are readily found in and invade freshwater habitats and we expect it should be challenging for *N. fasciata* to invade high-salinity environments (although we observed *N. fasciata* in salt marsh habitats). Nonetheless, a similar pattern is seen in Andean Speckled Teals (*Anas flavirostris*) where low-altitude populations migrate at a higher rate into high-altitude populations despite positive selection in hemoglobin genes within high-altitude populations (Graham et al. 2018). The most likely explanation for this pattern is a “source-sink” dynamic between lineages which might reflect the expansion or invasion of the larger and more widely distributed population, *N. fasciata*, into coastal habitats. Following invasion of coastal habitats, selection can favor individuals with adaptations to high-salinity environments leaving the remainder of the genome free to admix (Graham et al. 2018). Future study should focus on estimating the effective population sizes of these two species to elucidate potential “source-sink” dynamics; however, preliminary analysis of nucleotide diversity in these two species supports this hypothesis with significantly higher nucleotide diversity in *N. fasciata* (π = 0.29) relative to *N. clarkii* (π = 0.25; *P *<* *0.001). An alternate explanation may be exclusion, where *N. clarkii* alleles are less able to permeate into *N. fasciata* due to potential trade-offs with fitness and subsequent inability to compete with the dominant *N. fasciata* populations. Under this scenario, *N. fasciata* likely functions as a generalist capable of moving across the landscape and *N. clarkii* as a coastal specialist.

An ongoing challenge for watersnakes and many other groups with widespread gene flow is reconciling taxonomy with complex, reticulate evolutionary histories. Since the initial description of lineages within the *N. fasciata-clarkii* complex, the nomenclature has undergone frequent revision with lineages being listed as subspecies of the Northern Watersnake (*Nerodia sipedon*), then the Southern Watersnake (*N. fasciata*), and later being re-elevated to species or subspecies based on morphology, allozymes, and ecological differentiation (Carr and Goin 1942; Lawson et al. 1991; reviewed in Territo 2013). Although we confirm ecological divergence of *N. fasciata* and *N. clarkii* in Florida and document evidence indicative of local adaptation, we also show a clear lack of reproductive isolation and no clear trend of morphological variation corresponding to recognized taxonomy. Therefore, our data are inconclusive with regard to taxonomy and only support that the *N. fasciata-clarkii* complex represent a case of incipient speciation, falling within the speciation continuum which may eventually diverge or coalesce (De Queiroz 2007). An alternative but complementary interpretation of our results is that this complex represents a single species with local adaptation along the coasts; however, without additional sampling from across the range of the *N. fasciata*-*clarkii* complex and explicit delimitation of species including demography and timing of divergence, we choose to avoid premature nomenclatural changes (Hillis 2019, 2020). Specifically, the timing of gene flow among our populations is uncertain and the observed patterns of admixture and gene flow could be consistent with allopatry followed by secondary contact or by ongoing divergence with gene flow. Additional genomic data, sampling from across the range of the *N. fasciata-clarkii* complex, and fine-scale analyses are required to better understand the intraspecific structure and adequately assess taxonomy. Notably, however, the federally listed subspecies, *N. c. taeniata*, may have the lowest proportion of admixture of the *N. clarkii* subspecies, suggesting further work should be done to evaluate the evolutionary distinctiveness of this lineage. Together, with the results on ecological divergence, our results suggest that IBD explains subspecies or population structure, similar to what was previously found for *N. c. compressicauda* by Jansen et al. (2008). However, IBE likely explains species divergence with genomic regions resistant to gene flow and locally adapted to high-salinity environments. The *N. fasciata-clarkii* complex is but one of many representative groups which rejects the bifurcating speciation model in favor of a more reticulate pattern (Dean et al. 2019; Edelman et al. 2019; Mason et al. 2019; Choi et al. 2020). As studies documenting gene flow and reticulate evolution (even among established species) increase in frequency, it is critical to consider how to best address taxonomy and ultimately conservation.

Phylogenomics and novel methodologies testing for violations of the bifurcating tree model have allowed gene flow and reticulate evolution to be widely recognized as a common process in lineage diversification (Nosil 2008; Barth et al. 2020). Given these technological developments, evolutionary biologists are able to more easily identify the forces and processes underlying incipient speciation with gene flow and begin to reconcile this with taxonomy and nomenclature. Environmental divergence can facilitate selection for local adaptation creating regions in the genome resistant to gene flow, but leaving the remainder of the genome free to admix. This pattern of divergent genomic islands follows a model of genetic hitchhiking (Feder et al. 2012). However, if divergence does not continue and the migration-selection balance is stable, reconciling the high degree of admixture with taxonomy has considerable implications, particularly for conservation. Consequently, it is critical to identify the mechanisms and regions of the genome underlying lineage divergence and explore alternate evolutionary histories, including reticulate evolution, to understand the speciation process.

## Materials and Methods

### Sampling

We sampled *N. fasciata* and *N. clarkii*, including all subspecies, from throughout Florida; we attempted to encompass individuals and localities from each potential habitat. Snakes were captured via road cruising or surveys in suitable habitat throughout Florida. Upon capture, locality was recorded and tissue was either taken in the field—via tail-tips, scale-clips, or blood draws—or the specimen was processed, vouchered, and deposited following AVMA guidelines (Leary et al. 2013). Species and subspecies identity were recorded for each sample using a combination of geography and preliminary morphological examination, according to the original subspecies descriptions (Baird and Girard 1853; Kennicott 1860; Cope 1895). If identity was uncertain, samples were recorded as such; however, all analyses were performed using unbiased clustering methods. Specimens were deposited at the Florida Museum of Natural History under accessions given in supplementary table S1, Supplementary Material online. Snakes were handled and collected under the following permits and Animal Care and Use Protocols: University of Central Florida IACUC (09-28W, 11-41W, 14-26W, and 15-39W), Florida Fish and Wildlife Conservation Commission (LSV-09-0295), Florida Department of Environmental Protection Division of Recreation and Parks (#8261020) and the United States Fish and Wildlife Service (TE220923-D).

### Sanger Sequencing

To determine phylogenetic structure, we first amplified and sequenced the cytochrome b gene (*cytb*: Burbrink et al. 2000) for 268 individuals and 20 outgroups (supplementary table S1, Supplementary Material online). We then selected a subset of 85 individuals and 11 outgroup taxa for additional gene sequencing and analyses (supplementary table S1, Supplementary Material online). In addition to *cytb* for these individuals, we amplified and sequenced NADH dehydrogenase subunits 1 (*ND1*: Jiang et al. 2007) and 4 (*ND4*: Arévalo et al. 1994), the nuclear gene Prolactin Receptor (*PRLR*: Townsend et al. 2008), the anonymous loci *M* and *E* (Mcvay et al. 2015), and the nuclear intron *TATA* (Wood et al. 2011). DNA was extracted from tissue samples using either a Qiagen DNeasy Kit or Serapure beads following the procedure from Rohland and Reich (2012). Genes were amplified using standard polymerase chain reaction (PCR) following the referenced studies or were optimized for samples where there was no initial amplification. All sequences were generated using Sanger sequencing by either the University of Arizona Genetics Core or Eurofins Genomics.

We aligned genes using *MUSCLE* (Edgar 2004) and manually inspected. We generated gene trees for each locus and also carried out phylogenetic analyses on three concatenated data sets: 1) an alignment of all loci, 2) an alignment of mitochondrial loci, and 3) an alignment of nuclear loci. We generated maximum-likelihood and Bayesian inference phylogenetic trees using *RAxML* v.8.2.12 (Stamatakis 2014) and *MrBayes* v.3.2.7 (Ronquist et al. 2012), respectively. In all analyses, protein-coding loci were partitioned by codon position and models of substitution were determined with *PartitionFinder* v. 2.1.1 (Lanfear et al. 2016). See supplementary table S2, Supplementary Material online, for alignment partitions and models. *RAxML* trees were generated with 20 independent searches with the substitution models set to GTR-GAMMA for all alignments. We annotated the resulting best tree with nodal support values attained by performing 1,000 bootstrap replicates. *MrBayes* analyses were run two independent times with one cold and three heated chains. Priors were set to program defaults with a variable rate prior across partitions. Chains were run for 10 million generations, sampling every 1,000 generations and the first 10% of samples were discarded as burn-in. Stationarity of the chain was evaluated using *Tracer* (Rambaut et al. 2018). All trees were viewed in *FigTree*.

### Double digest restriction-site associated DNA sequencing

We extracted DNA from blood, liver, or scale tissue using a standard phenol-chloroform extraction on 131 snakes. These individuals were chosen to encompass the variation observed within these taxa and individuals representing each of the described species/subspecies. We used a Qubit Fluorometer 3 (Thermo Fisher Scientific, Waltham, MA) to quantify extractions, requiring 500–1,000 ng of DNA for ddRADseq. Our ddRADseq protocol follows Peterson et al. (2012) with some modifications. Briefly, we digested DNA for a minimum of 8 h at 37 °C using the rare cutting restriction enzyme SbfI-HF (8 bp) and the common cutting restriction enzyme Sau3AI (4 bp). We purified samples using AMPure magnetic beads (Beckman Coulter, Inc., Irving, TX) at a 1.1:1 bead-to-DNA ratio and eluted in 42 μl of tris-HCl (pH 8.0) for quantification. Based on DNA concentration, we divided samples into groups of eight samples. To ensure similar coverage across samples, each group was assigned a standardized amount of DNA based on the lowest DNA concentration per group before ligation. One of 16 double-stranded DNA adaptors, or inline barcodes, was ligated to the end of the digested DNA fragments from each individual. These barcodes are necessary for sample identification after sequencing. Barcodes were ligated at 16 °C for 5 h. After ligation we cleaned each sample using AMPure beads at a 1.1:1 bead-to-DNA ratio and quantified DNA using Qubit. If necessary, samples were rearranged into new groups to minimize differences in DNA concentration. We pooled and cleaned groups with AMPure magnetic beads and eluted with 30 μl of TE (pH 8.0). We used a Pippin Prep (Sage Science) with a 1.5% agarose gel to size select fragments from 300 to 700 bp in length. This size-range was selected to recover the maximum number of potential loci for downstream analyses (Schield et al. 2015). The DNA fragments in each group were amplified using PCR following Schield et al. (2015). Each group was assigned a unique primer that adds a second index barcode to each sample during the PCR. This results in 128 unique barcode-index combinations. We cleaned samples twice at a 0.7:1 bead ratio and quantified on the Qubit. We confirmed concentration and library quality using a high-sensitivity DNA chip for the BioAnalyzer 2100 (Agilent Genomics) which confirm the appropriately sized fragments were amplified. We sequenced our libraries on an Illumina Nextseq 550 using 150 bp paired-end reads.

### Variant Calling

We used the *ipyrad* v0.7.28 (Eaton 2014; Eaton and Overcast 2020) toolkit to demultiplex reads, assemble loci, and call SNPs, with default parameters, unless otherwise specified. Briefly, each library had the first 8 bp trimmed using *Trimmomatic* v0.38 (Bolger et al. 2014) prior to demultiplexing to remove adapter/primer sequence. After demultiplexing, low-quality sites (Q < 20) were trimmed from the 3ʹ end of reads or changed to ambiguities (N). Reads with more than five ambiguities or that were less than 100 bp were removed. Reads were then clustered de novo within individuals using a 90% similarity threshold. Next, consensus allele sequences were estimated, considering an estimated sequencing error rate (0.003) and heterozygosity (0.02) across all sites. Only sites between 6 and 10,000× coverage and reads with less than 5 uncalled bases were used for genotyping. Using the same similarity threshold, consensus sequences were clustered across individuals and then aligned. Loci with more than eight heterozygous sites, or which occurred in less than four individuals, were removed.

We used *VCFtools* v0.1.15 (Danecek et al. 2011) to further filter SNPs. Individuals were removed if they had fewer than 1,000 loci. Next, in order to ensure high-quality SNPs, we removed any nonbiallelic SNPs as well as SNPs that had more than 50% missing data, a mean depth of less than 10× across individuals, and minor allele frequency greater than 5%. The depth flag was used to find the mean depth of each individual across all their reads and individuals below 5× were removed in order to remove individuals with low-quality data. This resulted in a final data set of 119 individuals (including the outgroup) with 2,260 loci containing 7,057 SNPs.

### Population Structure and Phylogenomics

First, we used a discriminant analysis of principal components, or *DAPC* (Jombart et al. 2010), in the *adegenet* package in R (R Core Team 2020) to identify distinct genetic clusters. The best number of clusters was identified with *k*-means clustering and models compared with Bayesian Information Criterion (BIC) for *K* = 1–20 with all principal components retained (supplementary fig. S2, Supplementary Material online). Models with a ΔBIC <2 were considered the best number of clusters. We then used *DAPC* to describe the clusters with the number of principal component axes retained chosen via cross-validation using 100 replicates and a training proportion of 0.9.

Additionally, we tested for population structure using the R package *conStruct* to dissociate population structure from continuous clines of genetic variation (Bradburd et al. 2018). First, vcf files were converted to *Structure* input files using *plink* (Chang et al. 2015) and a pairwise distance matrix among samples was generated. To test if spatially aware models were better fit than nonspatial models, we performed cross-validation for *K* = 1–7 with ten replicates per value of *K*, 100,000 iterations per replicate, and a training proportion of 0.9. Cross-validation demonstrated that spatially aware models always performed better and suggested IBD is a feature of our data (supplementary fig. S3, Supplementary Material online). However, in the cross-validation analysis, we found strong support for each increasing value of *K*. Therefore, to assess how much each additional layer (*K*) contributes to the explanation of the data, we also performed an analysis of layer contributions. Specifically, we ran a spatially aware construct analysis for *K* = 2–7 with three Markov chain Monte Carlo (MCMC) chains and 10,000 iterations per MCMC. Layer contributions from each model were then loaded and plotted (supplementary fig. S4, Supplementary Material online).

To infer a phylogeny from the SNP data, we ran *SVDquartets* (Chifman and Kubatko 2014) with a random sample of 500,000 quartets, 1,000 bootstrap replicates, and *T. saurita* set as the outgroup. Specifically, we converted SNPs to a nexus alignment with two alleles per individual. Lineage and species trees were inferred by partitioning based on the individuals (coalescing alleles from the same individual together) and the *DAPC* clusters, respectively. A majority rule consensus tree was inferred from the bootstrap replicates.

### Gene Flow

To test for evidence of gene flow among populations, we ran *Treemix* (Pickrell and Pritchard 2012) on the *DAPC* identified populations at *K* = 5, 6, and 7. Based on the results from *SVDquartets*, we rooted *Treemix* with the *N. f. fasciata* population and allowed 0–15 migration events with ten iterations per migration edge. To account for linkage disequilibrium and provide confidence in the population graph, we used bootstrap resampling in blocks of 1,000 SNPs (-k 1,000). To estimate the optimal number of migration edges for each value of *K*, we used the R package *OptM* (Fitak Forthcoming) using the default Evanno method. The number of migration edges was chosen based on a plateau of log likelihood and when greater than 99.8% of the variance was explained.

In addition, we used the program *HyDe* (Blischak et al. 2018) to detect hybrids and gene flow. Specifically, *HyDe* uses phylogenetic invariants arising under a coalescent model with hybridization. We ran *HyDe* using all possible combinations of triples (i.e., parent–hybrid relationships) based on the *DAPC* clusters at *K* = 5, 6, and 7. We then ran 200 bootstrap replicates on the significant *HyDe* results and plotted the resulting bootstrap values of gamma (ɣ) using *ggplot2* (Wickham 2016) and *ggridges* (Wilke 2020).

To determine if the direction of gene flow might be due to differences in effective population size, we calculated nucleotide diversity (π) for each species (*DAPC K* = 2) as an indicator of effective population size. Nucleotide diversity was calculated in *VCFtools* and compared using a *t*-test in R.

To visualize inferred migration rates in the *N. fasciata-clarkii* complex in geographic space, we used the program *EEMS* (Petkova et al. 2016). *EEMS* estimates effective migration by visualizing regions where genetic dissimilarity decays quickly. It relates effective migration rates to expected genetic dissimilarities to define spatial features of population structure across the landscape. We estimated gene flow with *EEMS* across Florida by running three independent chains, each with 1,000 demes, for 2,000,000 MCMC iterations, with 500,000 iterations of burn-in and a thinning interval of 1,000. We plotted the results to check for agreement across all three chains and checked for MCMC convergence using the R package, *rEEMSplots*. After failing to converge, we restarted each chain for another 1,000,000 MCMC iterations with 0 burn-in after which the chains converged.

### Ecological Niche Divergence

To assess ecological niche divergence between *N. fasciata* and *N. clarkii*, we downloaded 7,320 occurrence records from GBIF (1,216 *N. clarkii* and 6,104 *N. fasciata*; GBIF.org 2019). Records outside of Florida were removed and further cleaned by removing points outside of the distribution for each species obtained from IUCN resulting in a final data set of 3,623 records (649 *N. clarkii* and 2,974 *N. fasciata*). For the input environmental data, we used the BioClim data set from the WorldClim database with spatial resolution of 30 arcsec (Hijmans et al. 2005), cation exchange capacity and pH of soils from the SoilGrid database at a depth of 5 cm and with a spatial resolution of 30 arcsec (Hengl et al. 2017), and landcover data from the EarthEnv database with a spatial resolution of 30 arcsec (Tuanmu and Jetz 2014). Importantly, cation exchange capacity of soil is highly correlated with salinity and used as a proxy for salinity (Saidi 2012). Environmental variables were reduced to only variables which contributed greater than 5% to the model for either species. Next, we used *Humboldt* to perform NOT and NDT using corrected e-space across the full distribution and shared e-space, respectively (Brown and Carnaval 2019). *Humboldt* found a nonsignificant equivalency statistic (*P *>* *0.05) for both the NOT and NDT and mixed significance for the background statistics (supplementary fig. S6, Supplementary Material online). These results suggest that the niches between these two species might be equivalent (Schoener’s *D* = 0.37); however, the tests are inconclusive and prone to Type I error (supplementary fig. S6, Supplementary Material online). To further explore this, we calculated the potential niche truncation index (PNTI; Brown and Carnaval 2019) and found high potential niche truncation for *N. fasciata* (PNTI >0.73). In other words, because *N. fasciata* occupies nearly all of Florida and *N. clarkii* is near the edge of available environmental space (i.e., coastal habitats), *Humboldt* is unable to sample appropriate background data to provide conclusive results.

To circumvent these issues, we looked for differences in the most important environmental variables identified by *Humboldt* for both GBIF data and assigned genetic clusters by extracting environmental data for each point and comparing species with *ggpubr* (Kassambara 2019). In addition, we performed linear regression of *conStruct* admixture proportion (i.e., proportion assigned to *N. fasciata*) and cation exchange capacity using *ggpubr*. Finally, we performed Mantel and partial Mantel tests to disentangle the influence of IBD and IBE caused by salinity. Specifically, we tested for correlations between genetic dissimilarity (as calculated for *EEMS*), geographic distance (km), and environmental distance (calculated as the Euclidian distance between each samples value for cation exchange capacity). To test for IBD, we performed a Mantel test between genetic dissimilarity and geographic distance. To test for IBE, we performed a partial Mantel test between genetic dissimilarity and environmental distance, controlling for geographic distance. These tests were performed on three data sets: 1) all individuals, 2) *N. fasciata* only (*DAPC K* = 2), and 3) *N. clarkii* only (*DAPC K* = 2).

### Scans for Selection Signatures

To test for signatures of selection between *N. fasciata* and *N. clarkii*, we searched for outlier SNPs using *BayeScan* (Foll and Gaggiotti 2008), blasted outlier loci to a reference genome to identify nearby genes, and identified enriched GO terms. First, we subset our data to only include *N. clarkii* individuals with <50% admixture with *N. fasciata*. Our final data set included 101 individuals (60 *N. clarkii* and 41 *N. fasciata*). The resulting data were converted to *BayeScan* format with *PGDSpider* (Lischer and Excoffier 2012). We ran *BayeScan* using default settings (i.e., 5,000 output iterations, 20 pilot runs, pilot run length of 5,000, 50,000 iterations of burn-in, and a thinning interval of 10). The loci with SNPs that were identified as significant (*P *<* *0.05; *n *=* *62 SNPs and 41 loci; supplementary table S3, Supplementary Material online) were blasted against the *T. elegans* genome (GCF_009769535.1) using *blastn* returning a maximum of five hits and hsps with a stringent *e*-value of 1e−50 (supplementary table S4, Supplementary Material online). We converted BLAST hits to a bed file (https://github.com/nterhoeven/blast2bed, last accessed 2020-10-18) and genes that were within 2,000 bp of a hit within the genome were extracted as potential genes of interest. To search for enriched GO terms, we used *ShinyGO* (Ge et al. 2019) using the *Gallus gallus* genome, an FDR cutoff of 0.05, and extracting the 30 most significant terms. We chose to use the chicken genome as our GO reference because it is the most closely related and complete database. Other reptilian databases (e.g., Anole) are less complete and do not have functional information matching many of the identified genes. We used the R package *igraph* (Csárdi and Nepusz 2006) to plot networks and identify modules with dense connections (cluster_edge_betweenness). Finally, we used the R package *circlize* (Gu et al. 2014) to plot connections of genes and enriched GO terms.

### Morphology

We counted or measured a total of 11 meristic and dimensional features for 137 specimens representing 24 *N. clarkii* (8 *N. c. clarkii*, 7 *N. c. compressicauda*, and 9 *N. c. taeniata*), 95 *N. fasciata* (21 *N. f. fasciata* and 71 *N. f. pictiventris*), and 21 with uncertain assignment to a taxon. Of the 11 features, we measured several diagnostic characters including the number of ventral scales, subcaudal scales, and tail length (Carr and Goin 1942). We measured snout-vent length to standardize tail length and also counted mid-body scale row. Lastly, we counted the number of supralabial scales, infralabial scales, and postocular scales on the left and right side of the body independently. To visualize morphological variation, we performed PCA with 95% confidence ellipses in R with the packages *ggpubr*. We used the first two principal component axes to perform model-based clustering analysis to identify the most likely clusters using the R package *mclust* (Scrucca et al. 2016). To determine what factors best explain the differences in PC1 and PC2, we created a set of linear models in R (R Core Team 2020) using the species assignment, subspecies assignment, sex, age, and capture latitude as explanatory variables and compared the models using AIC with the package *bbmle* (Bolker and R Development Core Team 2019). Species and subspecies assignment were based on the assignment at capture (*n *=* *137) and when only using samples with corresponding genetic data (*n *=* *21) we used *DAPC* assignment and *conStruct* admixture proportion to assess agreement between morphological and genetic data sets. We also performed a Mantel test between genetic dissimilarity (as calculated for *EEMS*) and the Euclidian distance of PC1.

## Supplementary Material

Supplementary data are available at *Molecular Biology and Evolution* online.

## Supplementary Material

msaa266_Supplementary_Data
