# Supplementary material for: Genomic Adaptations to Salinity Resist Gene Flow in the Evolution of Floridian Watersnakes
Source: Mol Biol Evol. 2020 Oct 9;38(3):745–60. doi: 10.1093/molbev/msaa266 (PMC7947766; doi:10.1093/molbev/msaa266)
Supplement: msaa266_Supplementary_Data [file msaa266_supplementary_data.zip › SupplementalFigures_v2.pdf]

Supplemental Figures

Fig. S1. BIC scores from k-means clustering of *DAPC* models for K=1–20 demonstrating that K=6 is the best supported model followed closely by K=5 and K=7.

Fig. S2. First two panels show cross-validation results of *conStruct* models for K=1–7. Increased support is shown for each additional layer (K) with spatial models (blue) outperforming nonspatial models (black). The second panel shows a clear increase in support at K=2 in the spatial models followed by a plateau in increased support. Third panel shows layer contributions for K=1–7 *conStruct* models showing that the first two layers contribute the most with each additional layer contributing very little.

Fig. S3. Single gene trees and concatenated mtDNA, nuDNA, and mtDNA+nuDNA phylogenetic analyses. Nodes less than 70% bootstrap support or posterior probability are collapsed.

S3.1. RAxML cytB phylogeny.
S3.2. MrBayes cytB phylogeny.
S3.3. RAxML concatenated mtDNA + nuDNA phylogeny. Genes include cytB, ND1, ND4, E, M, PRLR, TATA.
S3.4. MrBayes concatenated mtDNA + nuDNA phylogeny. Genes include cytB, ND1, ND4, E, M, PRLR, TATA.
S3.5. RAxML concatenated mtDNA phylogeny. Genes include cytB, ND1, ND4.
S3.6. MrBayes concatenated mtDNA phylogeny. Genes include cytB, ND1, ND4.
S3.7. RAxML concatenated nuDNA phylogeny. Genes include E, M, PRLR, TATA.
S3.8. MrBayes concatenated nuDNA phylogeny. Genes include E, M, PRLR, TATA.
S3.9. RAxML ND1 phylogeny.
S3.10. RAxML ND4 phylogeny.
S3.11. RAxML E phylogeny.
S3.12. RAxML M phylogeny.
S3.13. RAxML PRLR phylogeny.
S3.14. RAxML TATA phylogeny.

Fig. S4. *SVDQ*, *TreeMix*, and *HyDe* results for K=6 and K=7. *SVDQ* and *TreeMix* disagree in species tree topology likely due to tremendous introgression. At K=6, *TreeMix* infers three migration edges as the most likely network and *HyDe* infers five hybridization events. At K=7, *TreeMix* infers five migration edges as the most likely network and *HyDe* infers seven hybridization events.

Fig. S5. *HyDe* results indicating the hybrid origin of *N. f. pictiventris*, *N. c. compressicauda*, and *N. c. taeniata* as well as the parents of these clades. Bootstrap replicates indicate the distribution of gamma estimates for each significant result.

Fig. S6. *Humboldt* results including the Niche Overlap Test, Niche Divergence Test, and boxplots of the six most important environmental variables. NOT and NDT are inconclusive due to potential niche truncation, but *N. clarkii* tends to occupy habitats with more open water, regularly flooded vegetation, and higher salinity.

Fig. S7. *SVDquartets* inferred cladogram with bootstrap support values.

Fig. S8. Morphology of the *N. fasciata-clarkii* complex. (A) Sampling map of individuals with morphological data. (B–D) PCA of morphological data with (B) *mclust* inferred clusters and (C) colored by identification upon capture for species and (D) subspecies.

Fig. S9. (A) Morphological PC1 regressed against *conStruct* admixture proportions. (B & C) Violin plots demonstrating divergence in (B) PC1 between species and (C) PC2 between sexes given model support by AIC.

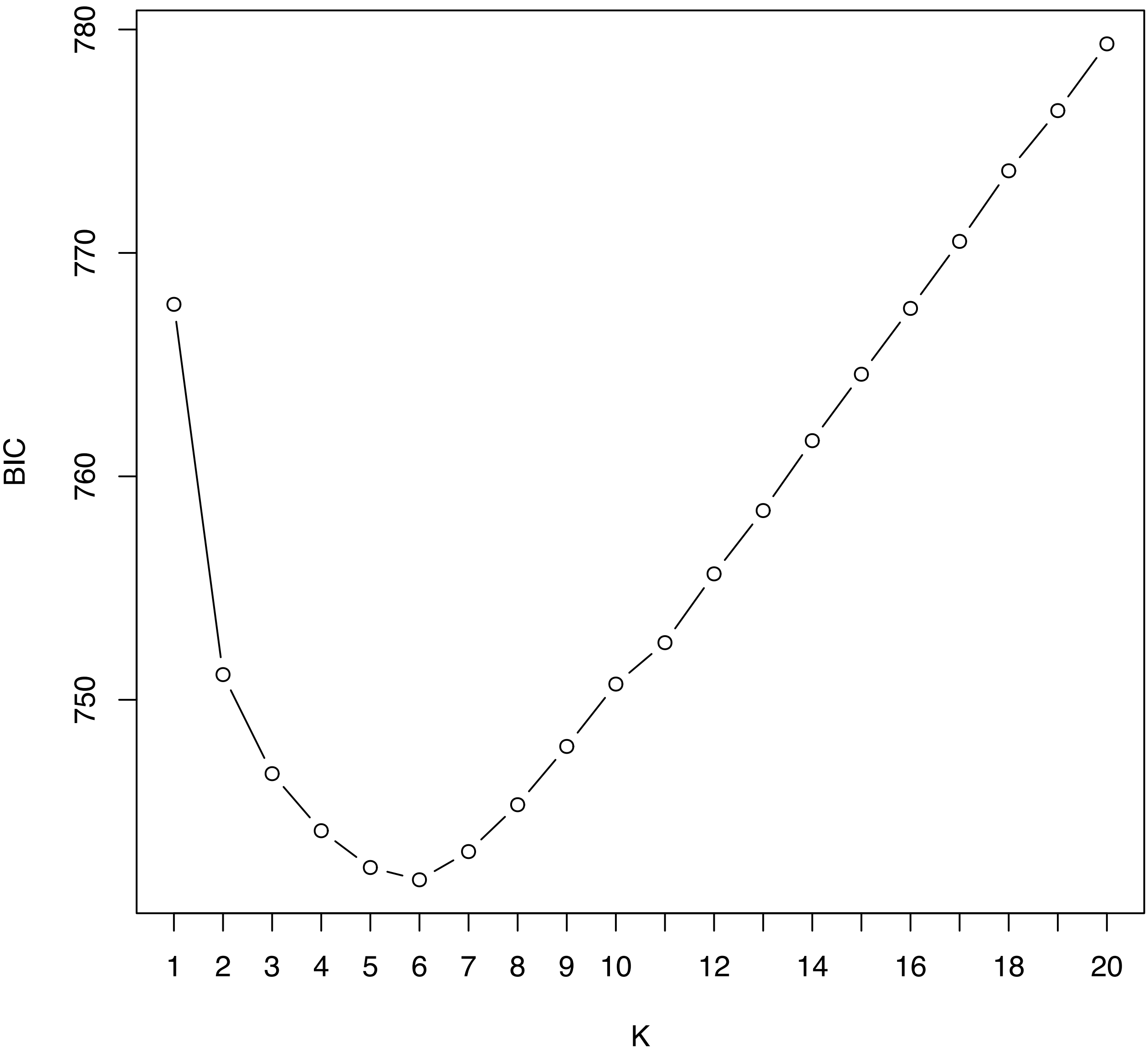

Fig S1. BIC scores from k-means clustering of *DAPC* models for K=1–20 demonstrating that K=6 is the best supported model followed closely by K=5 and K=7.

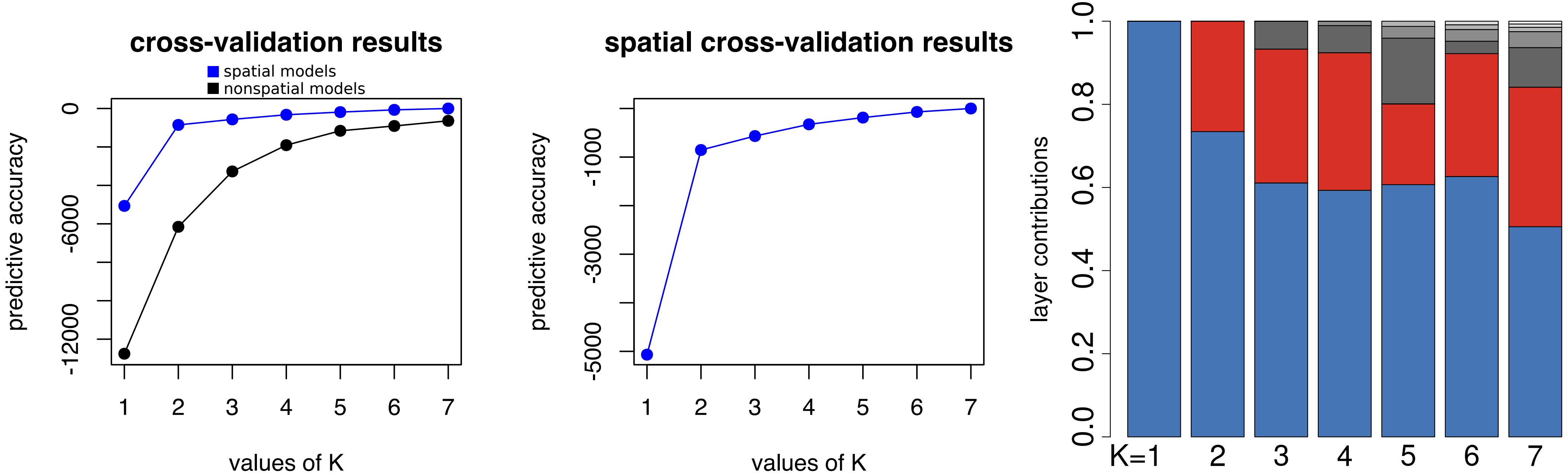

Fig S2. First two panels show cross-validation results of *conStruct* models for K=1–7. Increased support is shown for each additional layer (K) with spatial models (blue) outperforming nonspatial models (black). The second panel shows a clear increase in support at K=2 in the spatial models followed by a plateau in increased support. Third panel shows layer contributions for K=1–7 *conStruct* models showing that the first two layers contribute the most with each additional layer contributing very little.

S3.1. RAxML cytB phylogeny. Nodes less than 70% bootstrap support are collapsed.

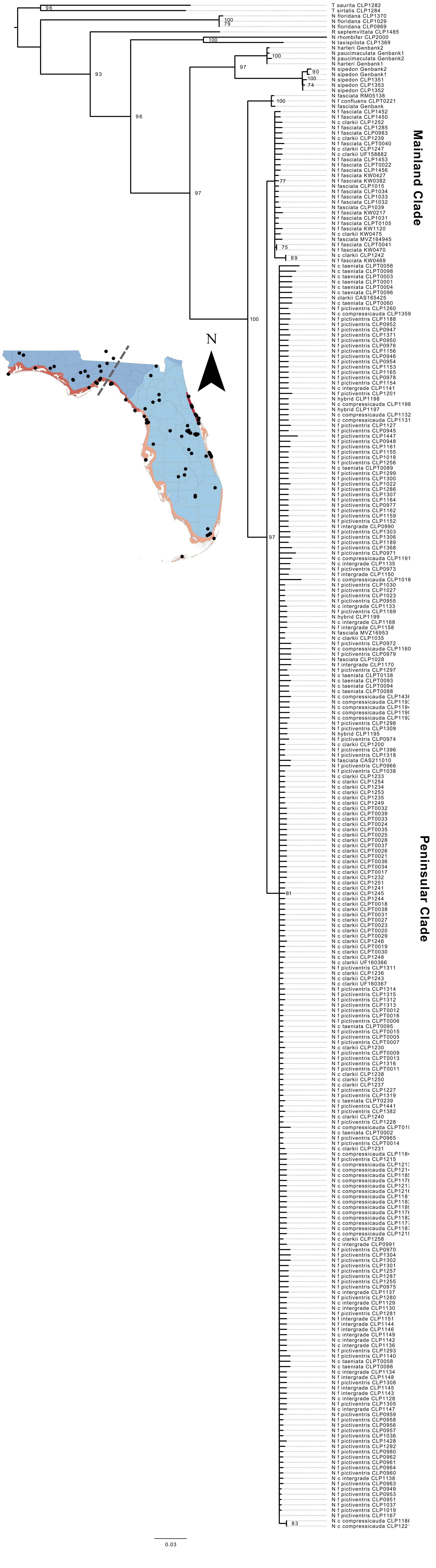

S3.2. MrBayes cytB phylogeny. Nodes less than 0.7 posterior probability are collapsed

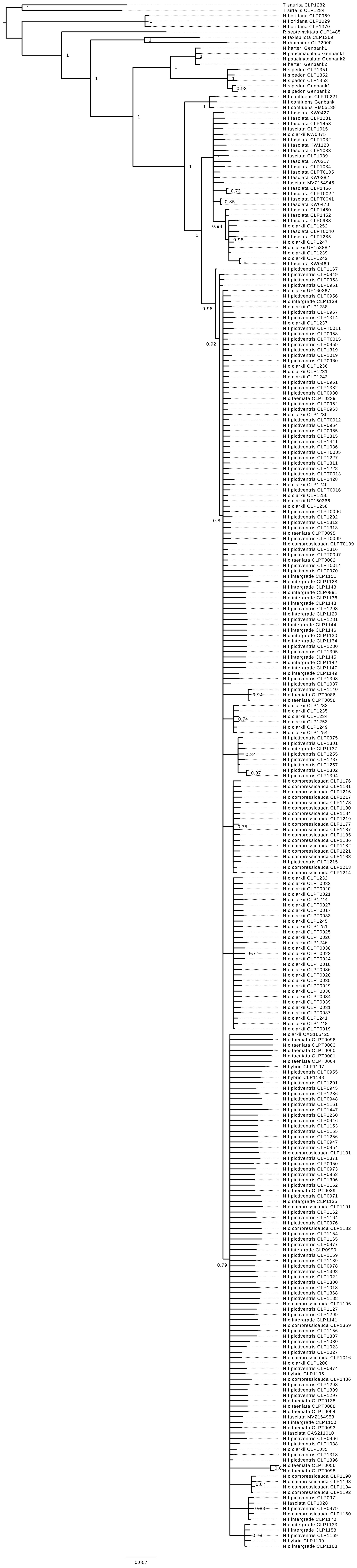

S3.3. RAxML concatenated mtDNA + nuDNA phylogeny. Genes include cytB, ND1, ND4, E, M, PRLR, TATA. Nodes less than 70% bootstrap support are collapsed.

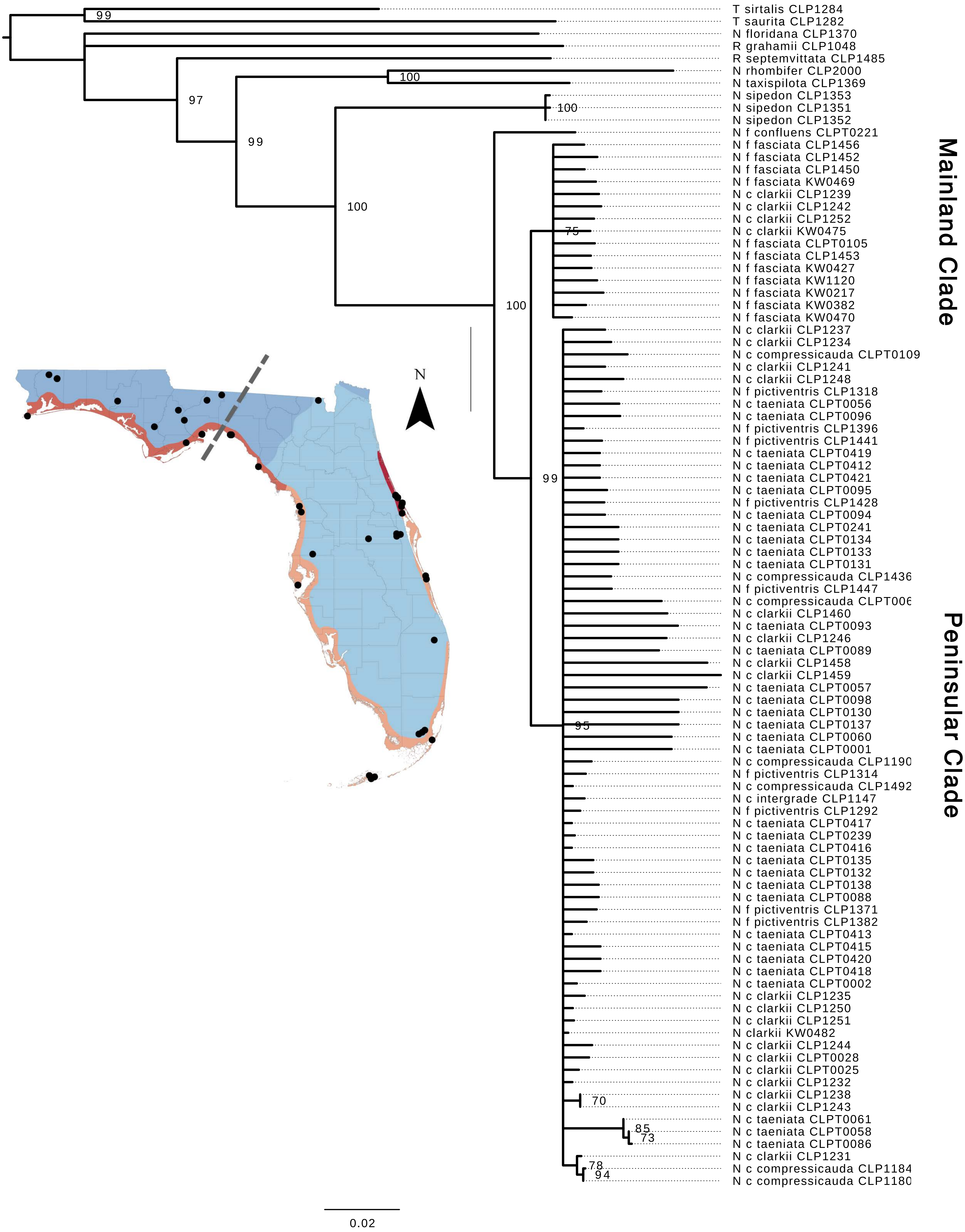

S3.4. MrBayes concatenated mtDNA + nuDNA phylogeny. Genes include cytB, ND1, ND4, E, M, PRLR, TATA. Nodes less than 0.7 posterior probability are collapsed.

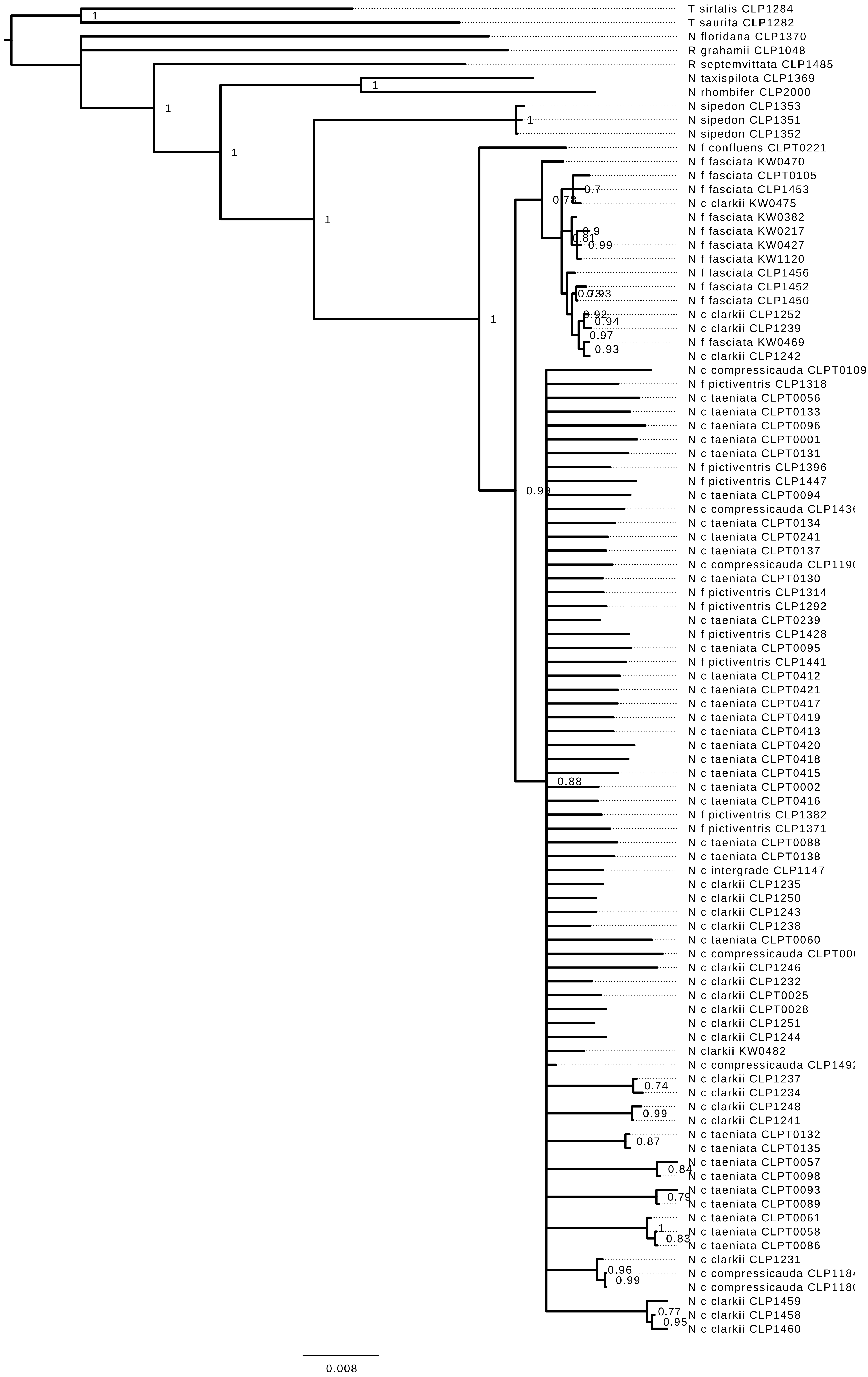

S3.5. RAxML concatenated mtDNA phylogeny. Genes include cytB, ND1, ND4. Nodes less than 70% bootstrap support are collapsed

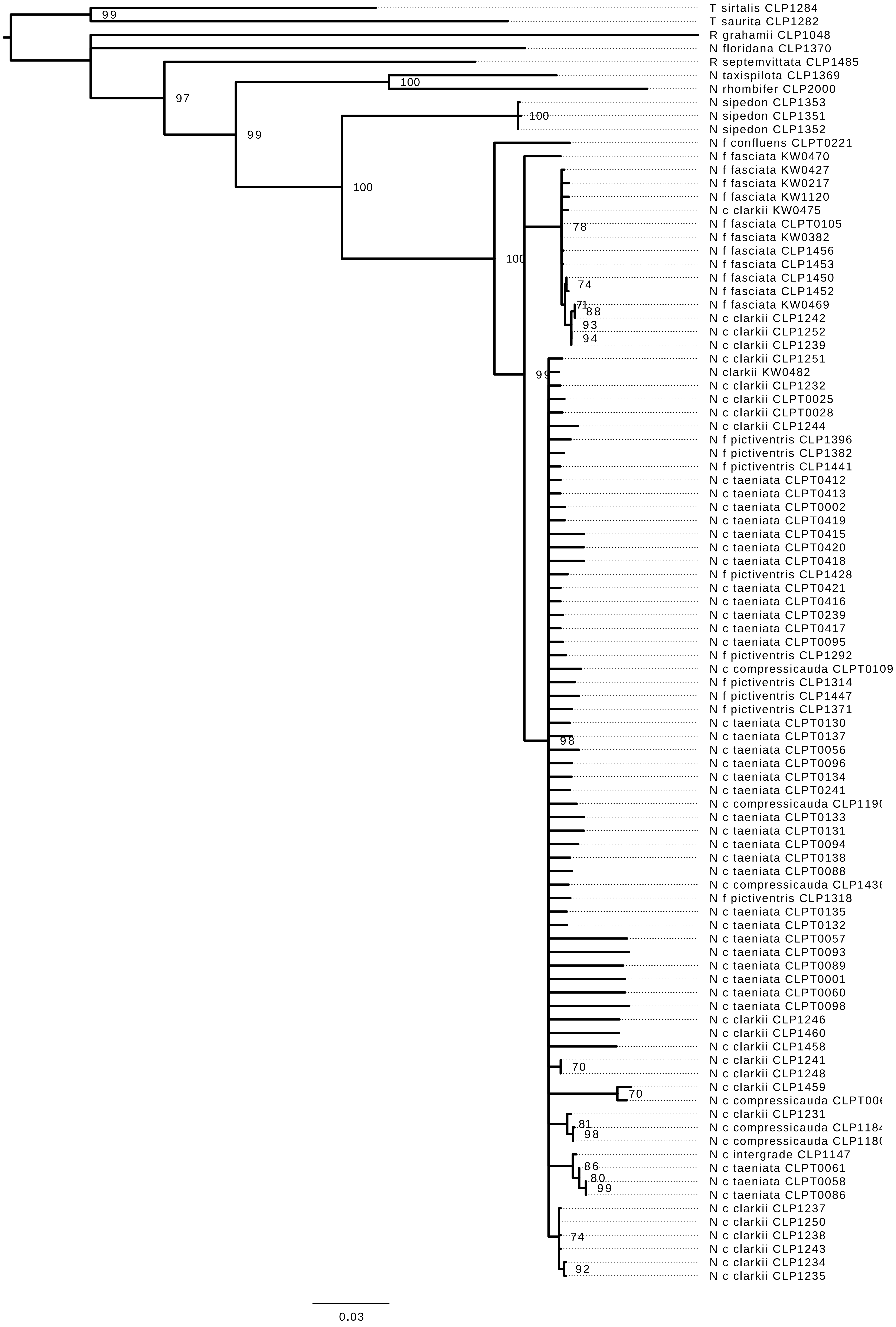

S3.6. MrBayes concatenated mtDNA phylogeny. Genes include cytB, ND1, ND4. Nodes less than 0.7 posterior probability are collapsed

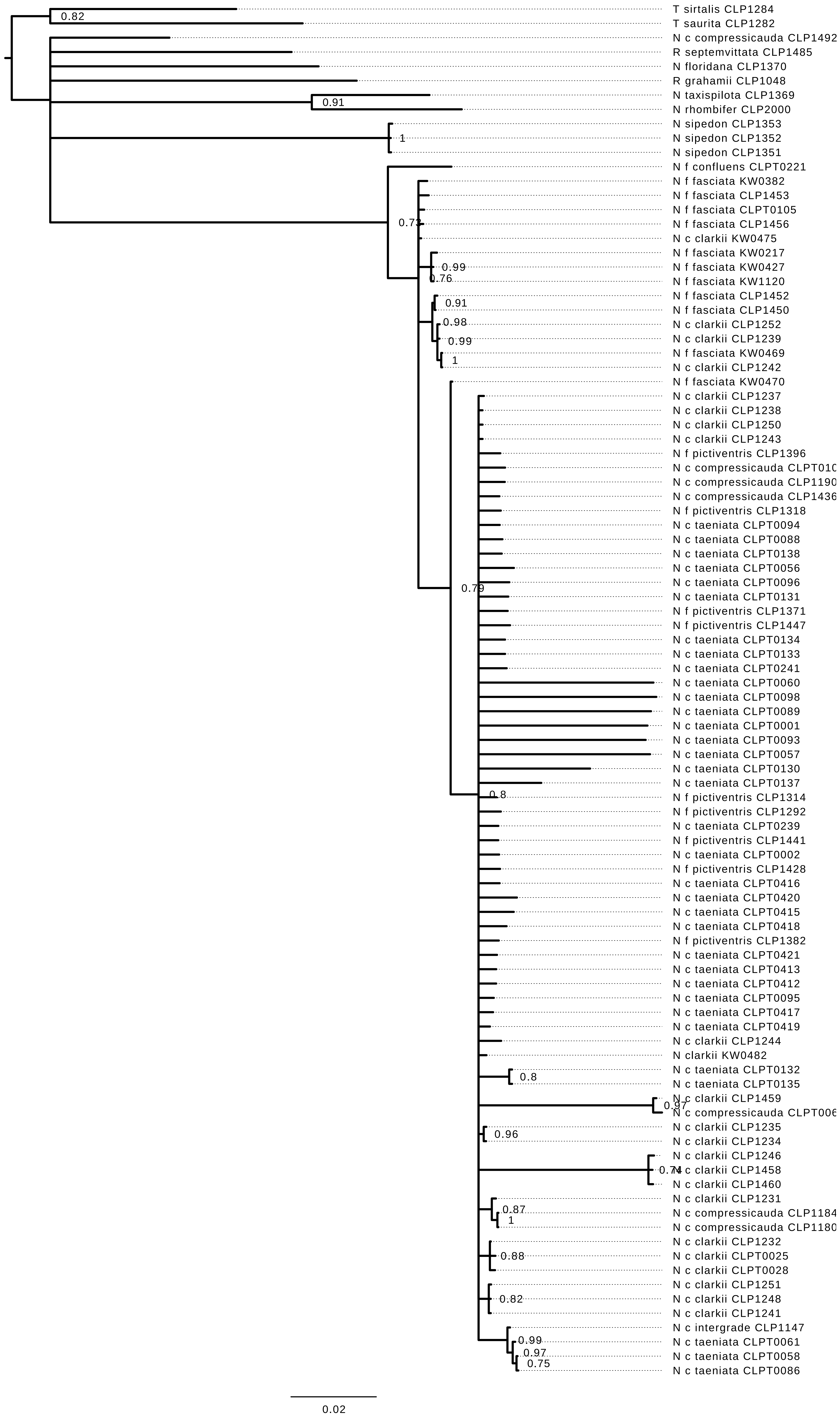

S3.7. RAxML concatenated nuDNA phylogeny. Genes include E, M, PRLR, TATA. Nodes less than 70% bootstrap support are collapsed

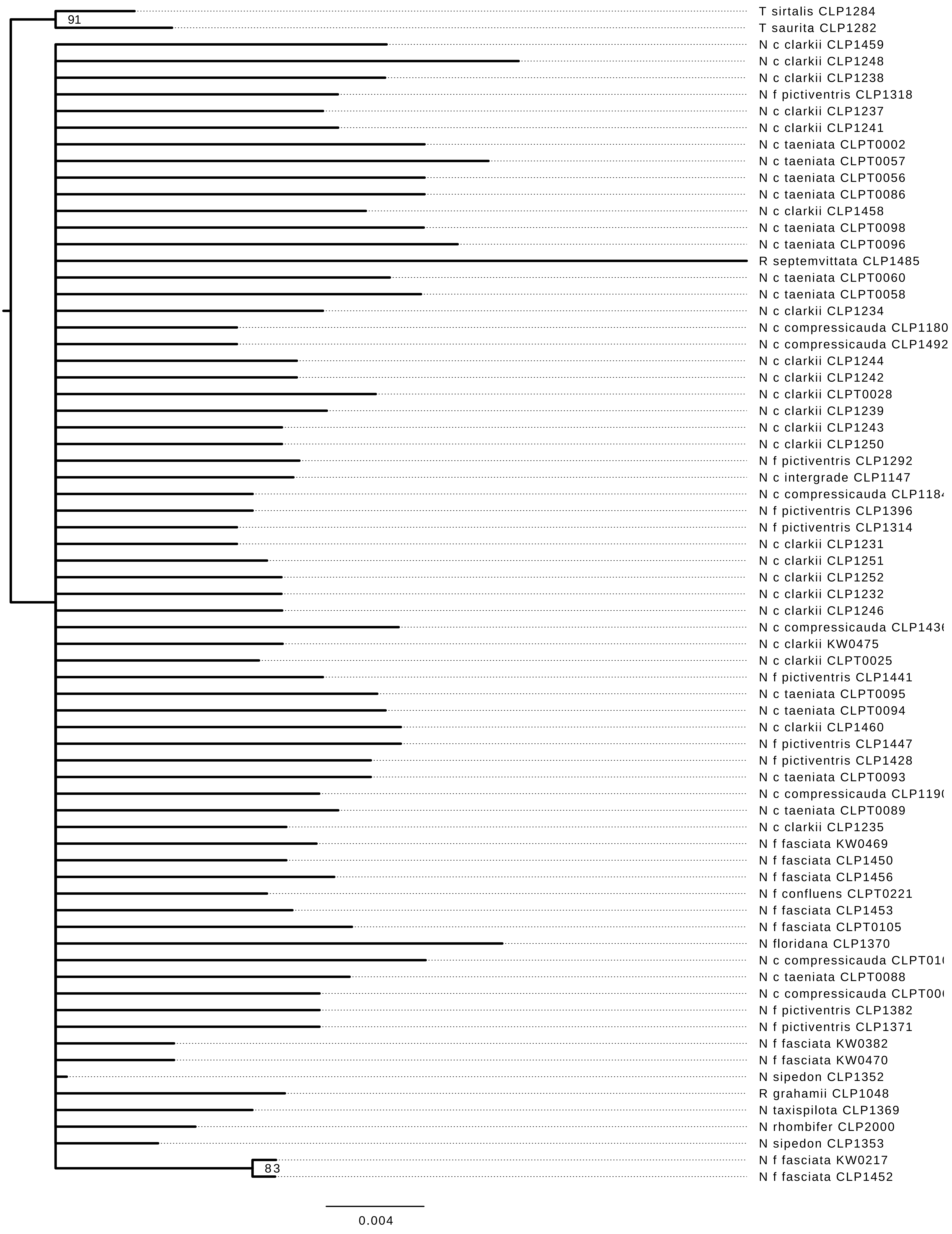

S3.8. MrBayes concatenated nuDNA phylogeny. Genes include E, M, PRLR, TATA. Nodes less than 0.7 posterior probabilty are collapsed

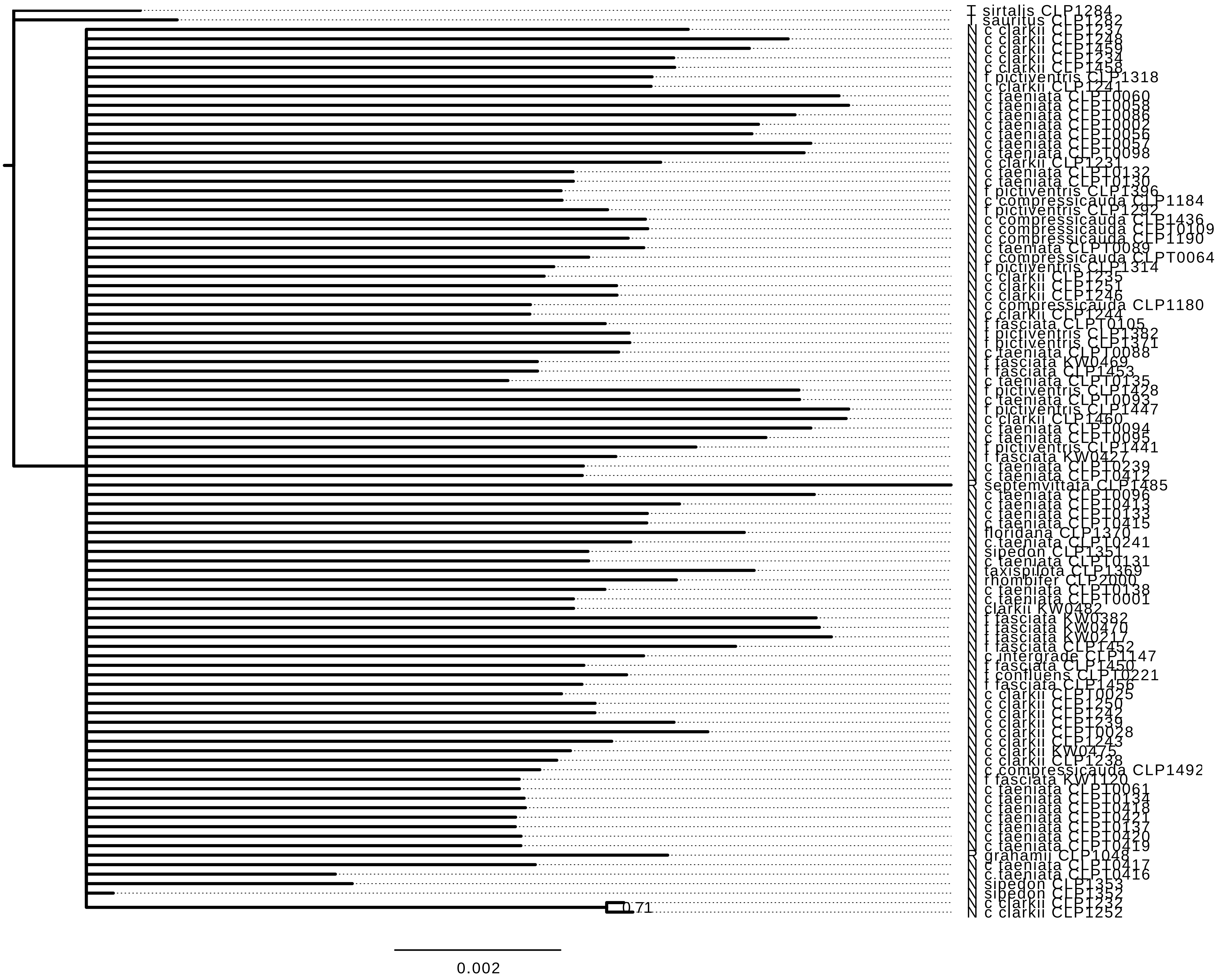

S3.9. RAxML ND1 phylogeny. Nodes less than 70% bootstrap support are collapsed.

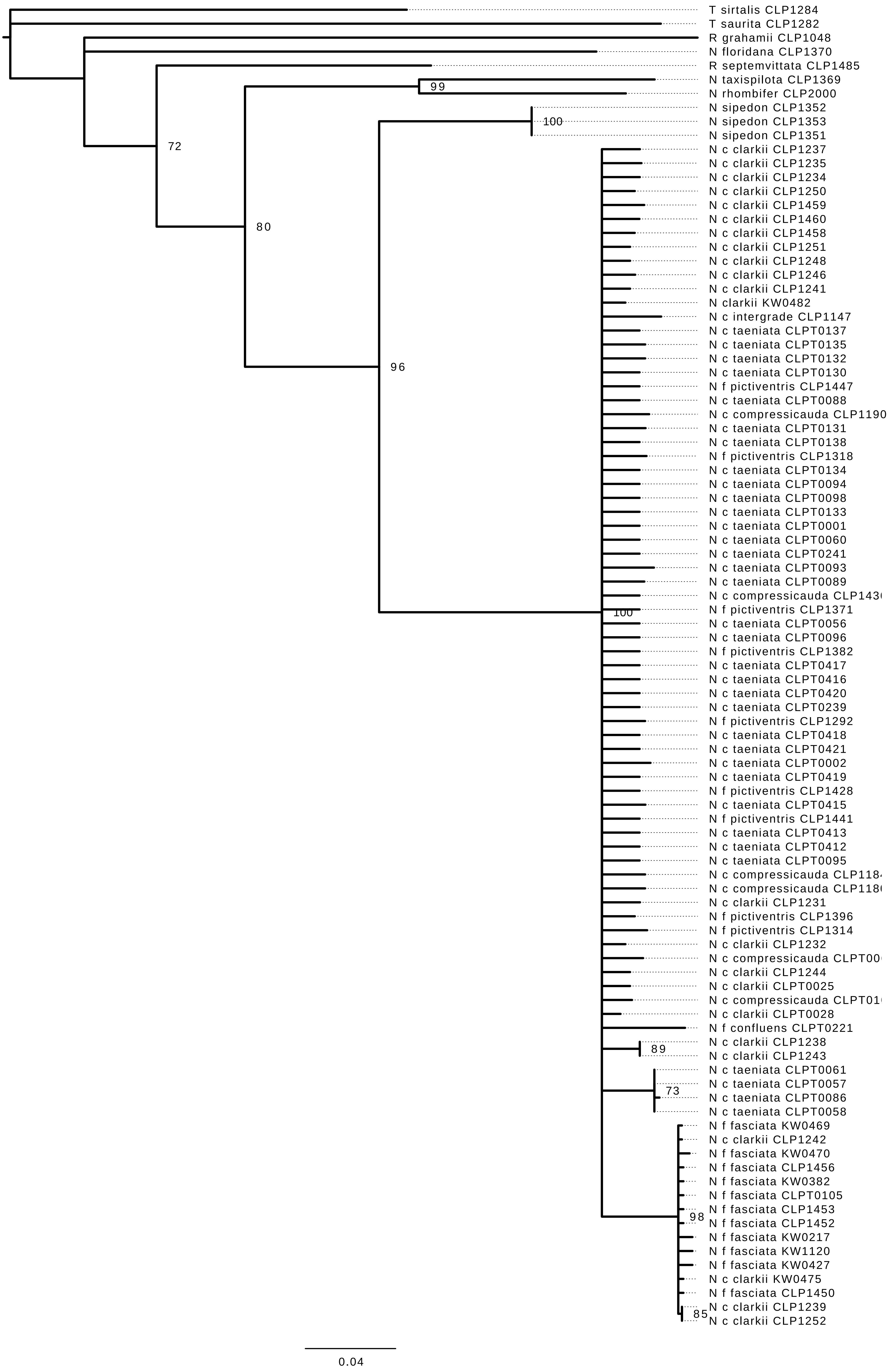

S3.10. RAxML ND4 phylogeny. Nodes less than 70% bootstrap support are collapsed.

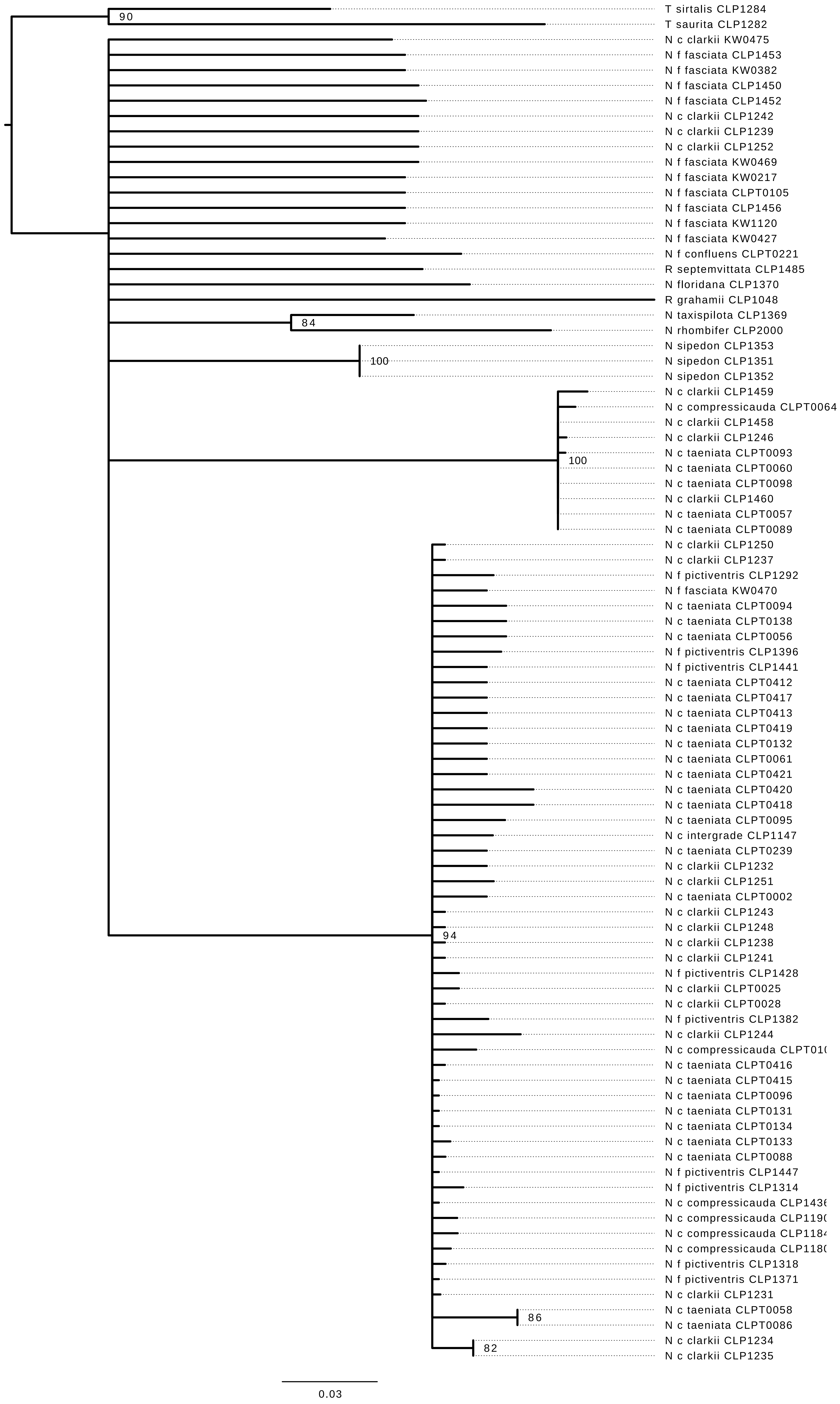

S3.11. RAxML E phylogeny. Nodes less than 70% bootstrap support are collapsed.

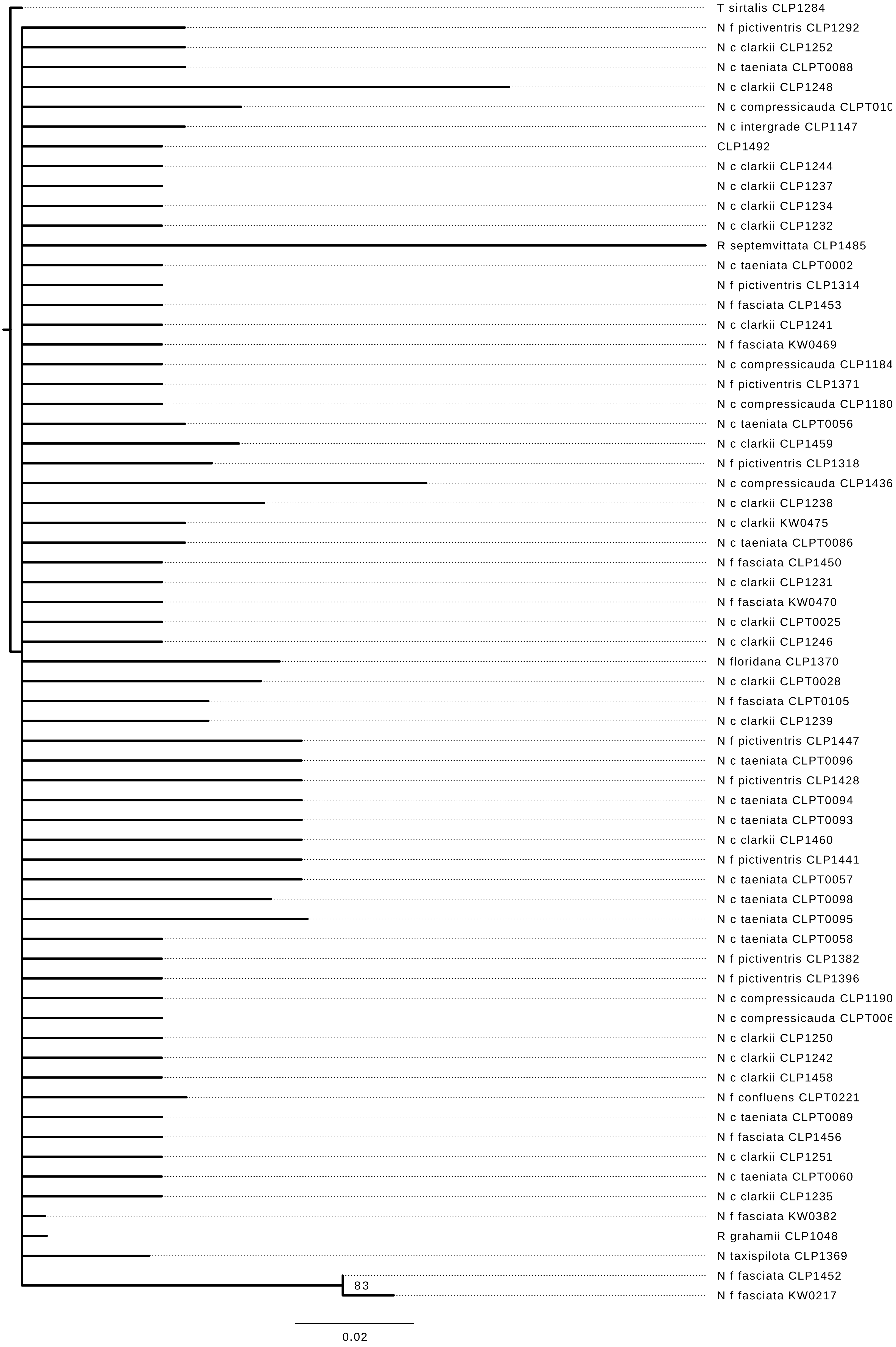

S3.12. RAxML M phylogeny. Nodes less than 70% bootstrap support are collapsed.

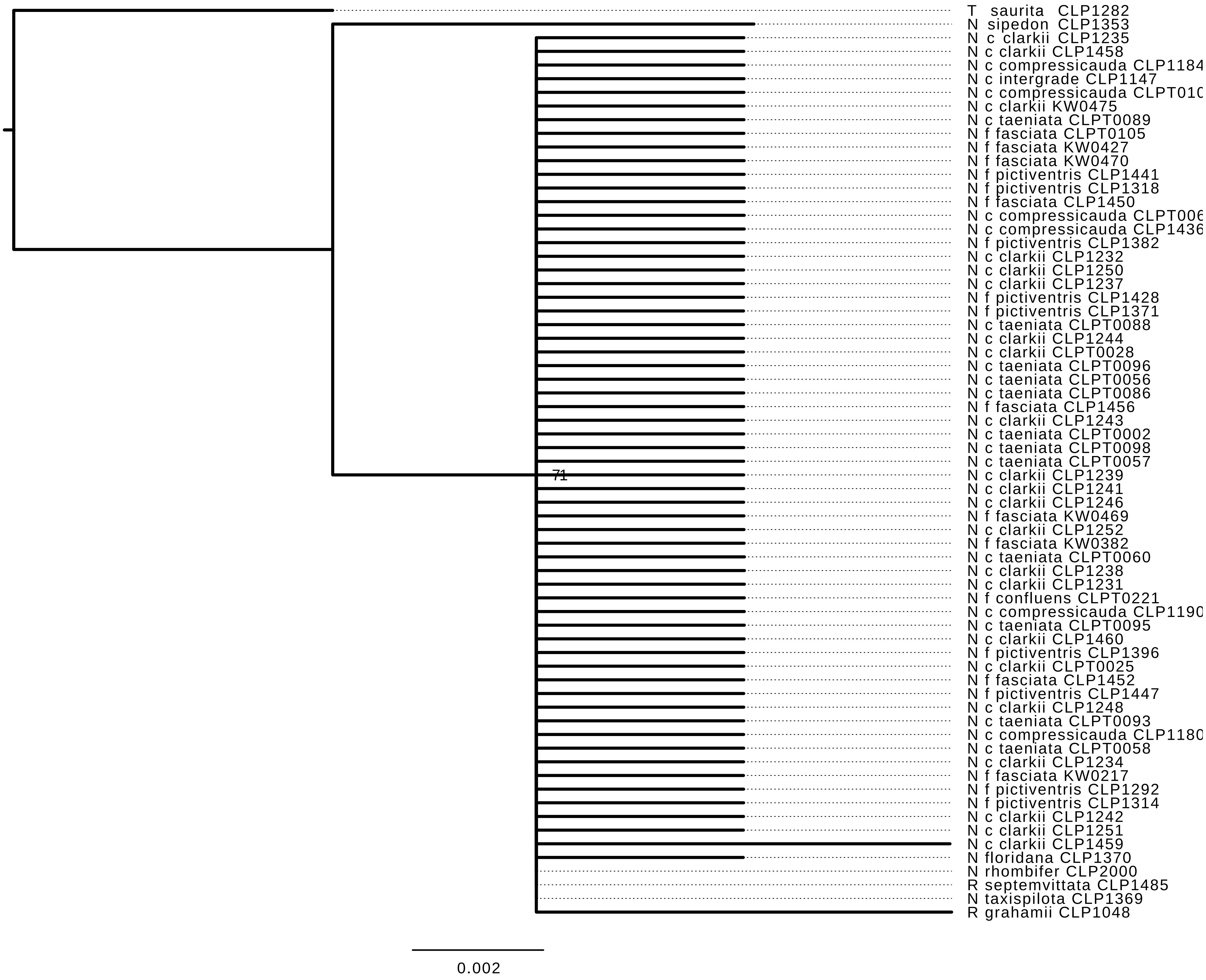

S3.13. RAxML PRLR phylogeny. Nodes less than 70% bootstrap support are collapsed.

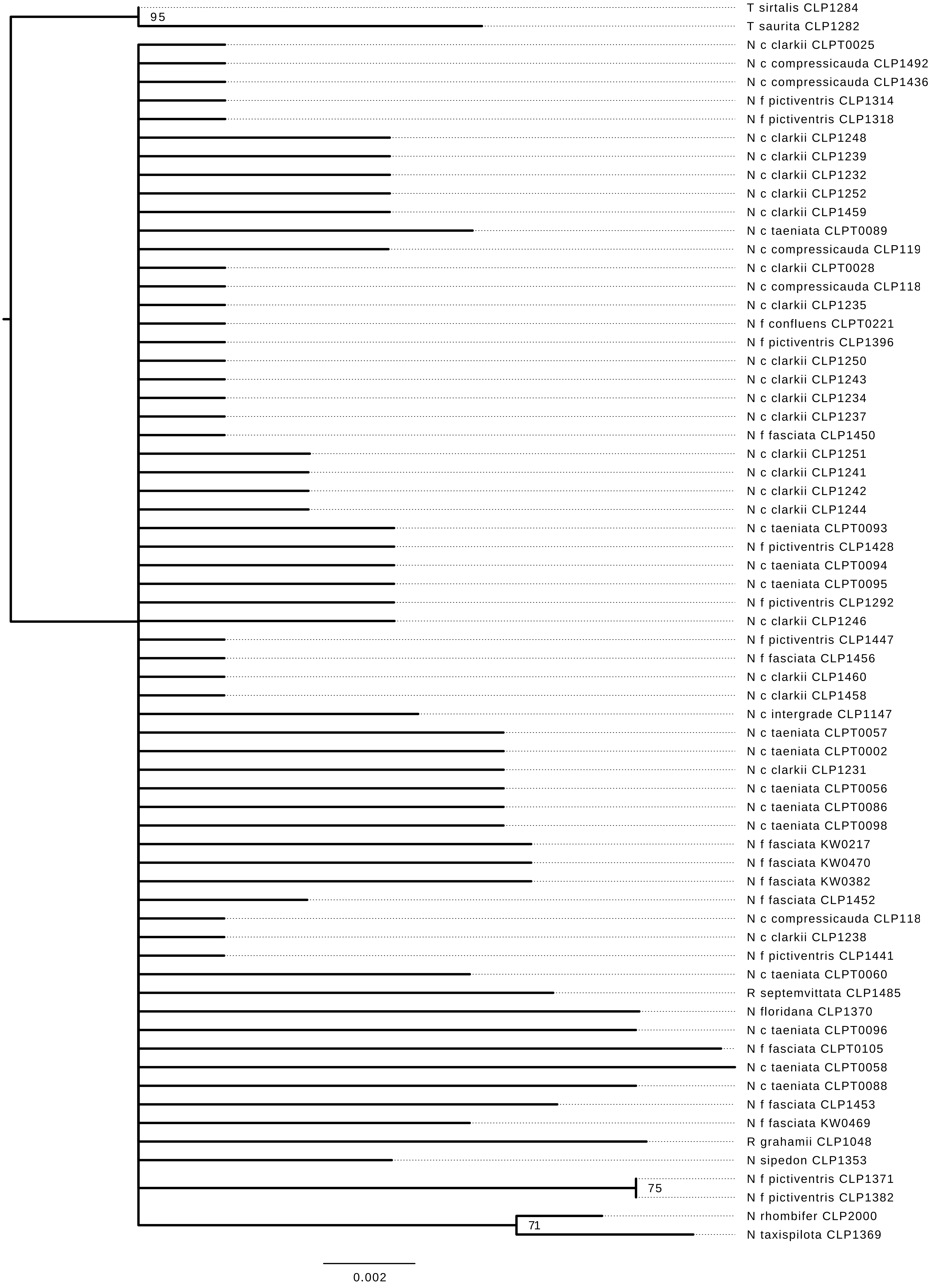

S3.14. RAxML TATA phylogeny. Nodes less than 70% bootstrap support are collapsed.

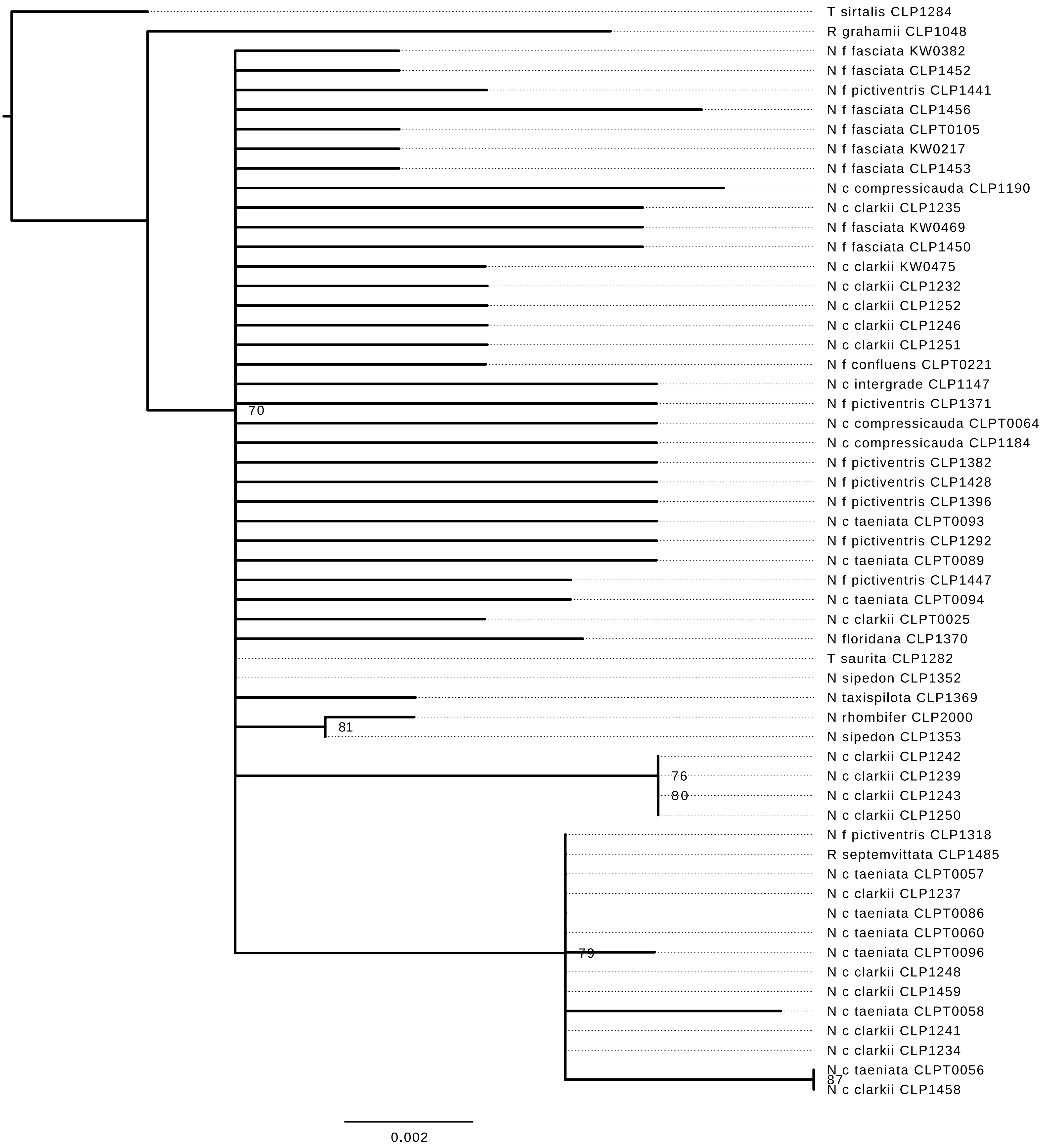

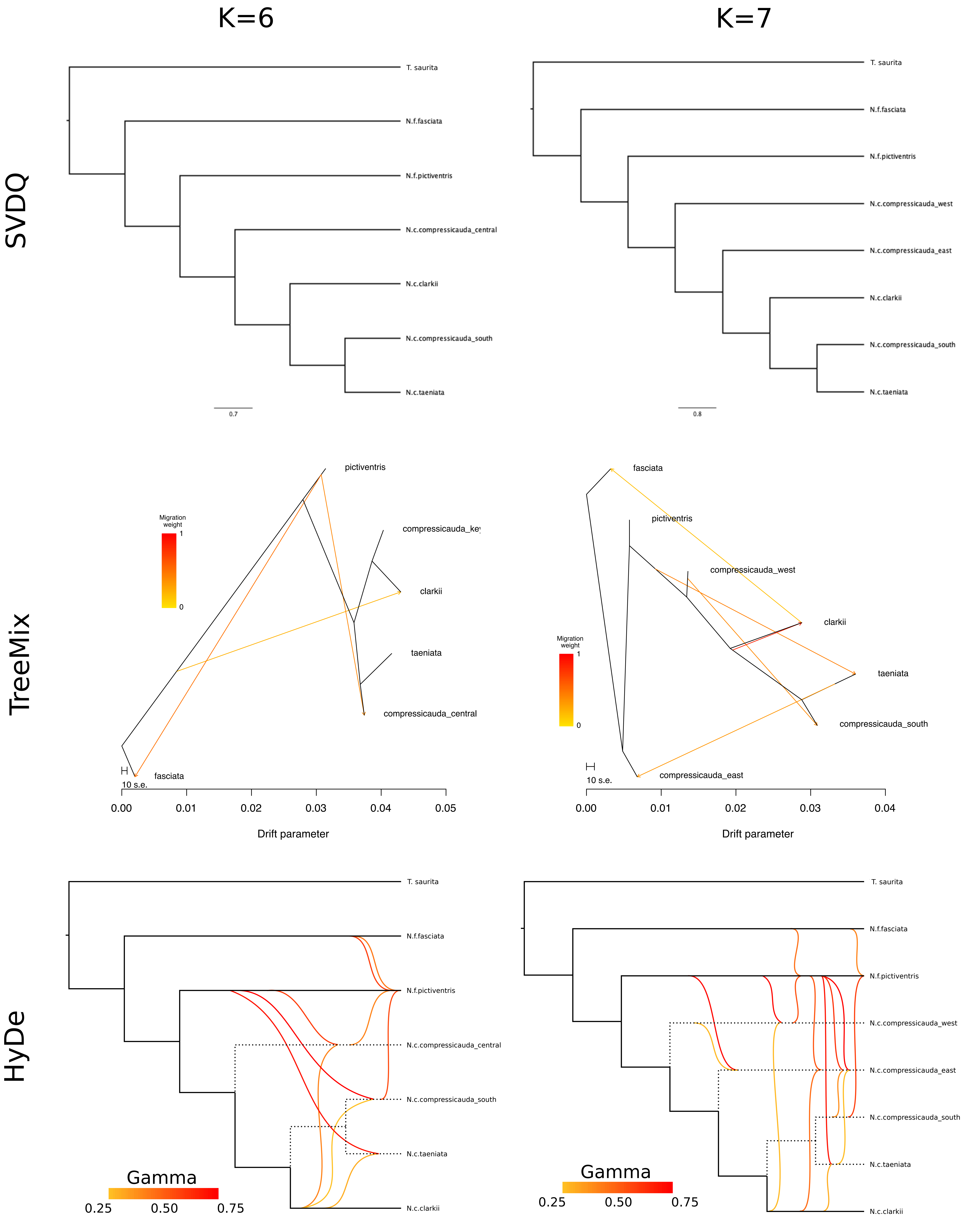

Fig S4. *SVDQ*, *TreeMix*, and *HyDe* results for K=6 and K=7. *SVDQ* and *TreeMix* disagree in species tree topology likely due to tremendous introgression. At K=6, *TreeMix* infers three migration edges as the most likely network and *HyDe* infers five hybridization events. At K=7, *TreeMix* infers five migration edges as the most likely network and *HyDe* infers seven hybridization events.

pops	P1	Hybrid	P2	Zscore	Pvalue	Gamma
	N_c_taeniata	N_f_pictiventris	N_f_fasciata	3.22	6.41E-04	0.49
dapc5	N_c_clarkii	N_c_compressicauda	N_f_pictiventris	4.79	8.39E-07	0.64
	N_c_clarkii	N_c_taeniata	N_f_pictiventris	3.62	1.49E-04	0.75
	N_c_compressicauda_eastwest	N_f_pictiventris	N_f_fasciata	3.18	7.29E-04	0.59
	N_c_compressicauda_south	N_f_pictiventris	N_f_fasciata	3.68	1.15E-04	0.44
dapc6	N_c_clarkii	N_c_compressicauda_eastwest	N_f_pictiventris	5.24	7.95E-08	0.59
	N_c_clarkii	N_c_compressicauda_south	N_f_pictiventris	3.25	5.79E-04	0.75
	N_c_clarkii	N_c_taeniata	N_f_pictiventris	3.91	4.64E-05	0.70
	N_c_compressicauda_south	N_f_pictiventris	N_f_fasciata	3.57	1.80E-04	0.41
	N_c_compressicauda_west	N_f_pictiventris	N_f_fasciata	3.65	1.29E-04	0.46
	N_c_clarkii	N_c_compressicauda_east	N_f_pictiventris	6.01	9.56E-10	0.53
	N_c_compressicauda_south	N_c_compressicauda_east	N_f_pictiventris	4.23	1.19E-05	0.62
dapc7	N_c_compressicauda_west	N_c_compressicauda_east	N_f_pictiventris	3.62	1.46E-04	0.73
	N_c_taeniata	N_c_compressicauda_east	N_f_pictiventris	3.37	3.74E-04	0.74
	N_c_clarkii	N_c_compressicauda_west	N_f_pictiventris	3.71	1.02E-04	0.73
	N_c_clarkii	N_c_taeniata	N_f_pictiventris	3.91	4.64E-05	0.70

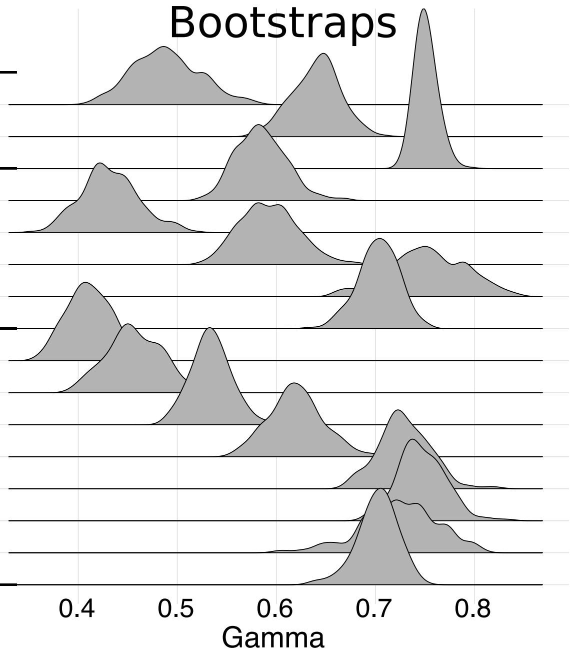

Fig S5. *HyDe* results indicating the hybrid origin of *N. f. pictiventris*, *N. c. compressicauda*, and *N. c. taeniata* as well as the parents of these clades. Bootstrap replicates indicate the distribution of gamma estimates for each significant result.

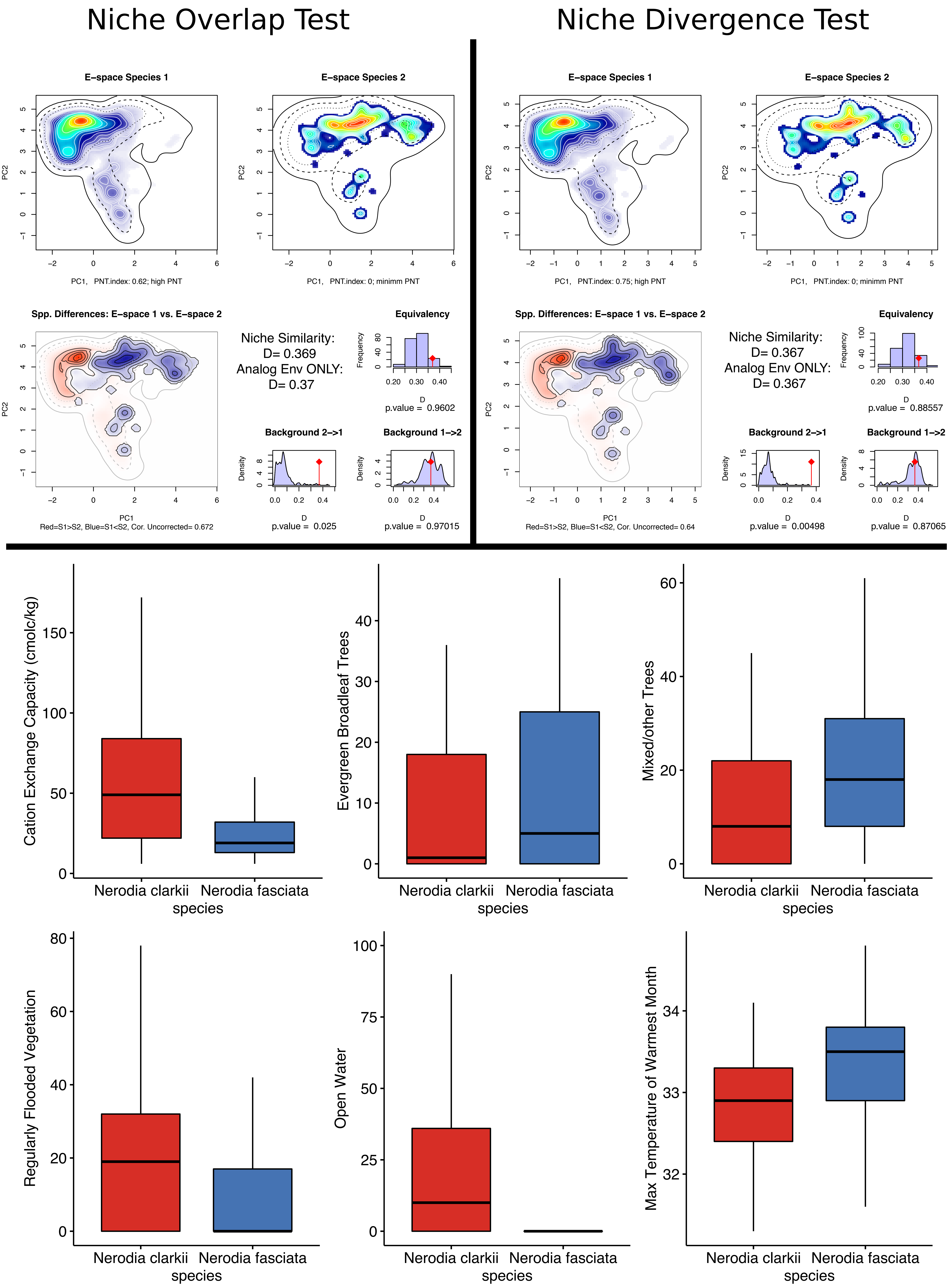

Fig S6. *Humboldt* results including the Niche Overlap Test, Niche Divergence Test, and boxplots of the six most important environmental variables. NOT and NDT are inconclusive due to potential niche truncation, but *N. clarkii* tends to occupy habitats with more open water, regularly flooded vegetation, and higher salinity.

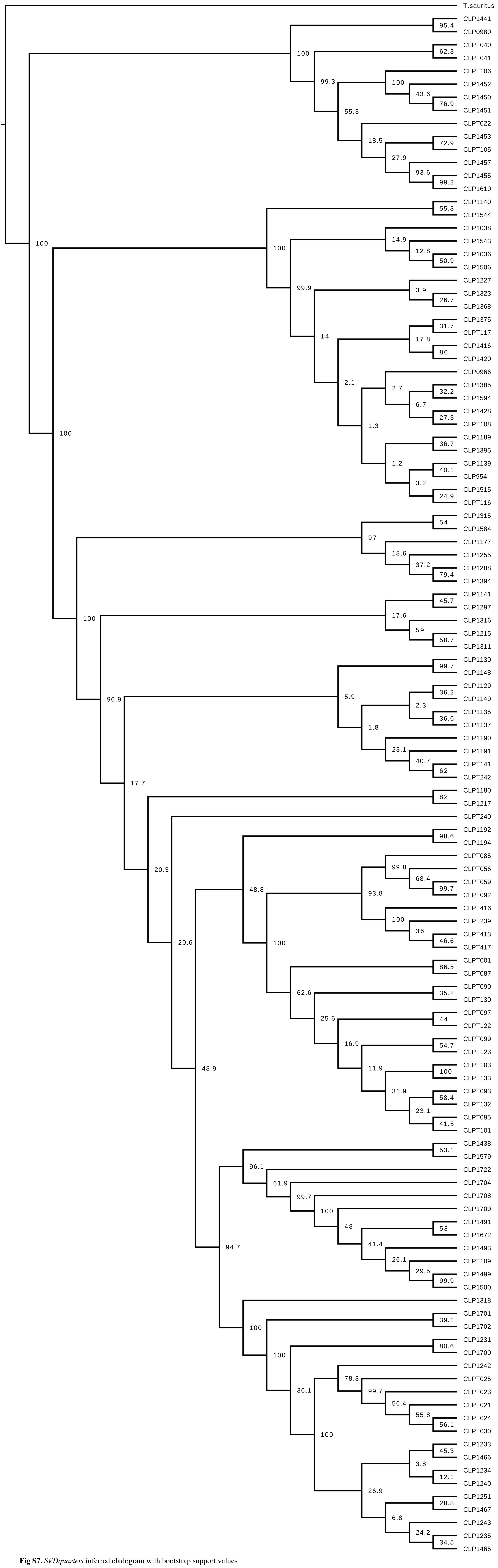

Fig S7. *SVDquartets* inferred cladogram with bootstrap support values

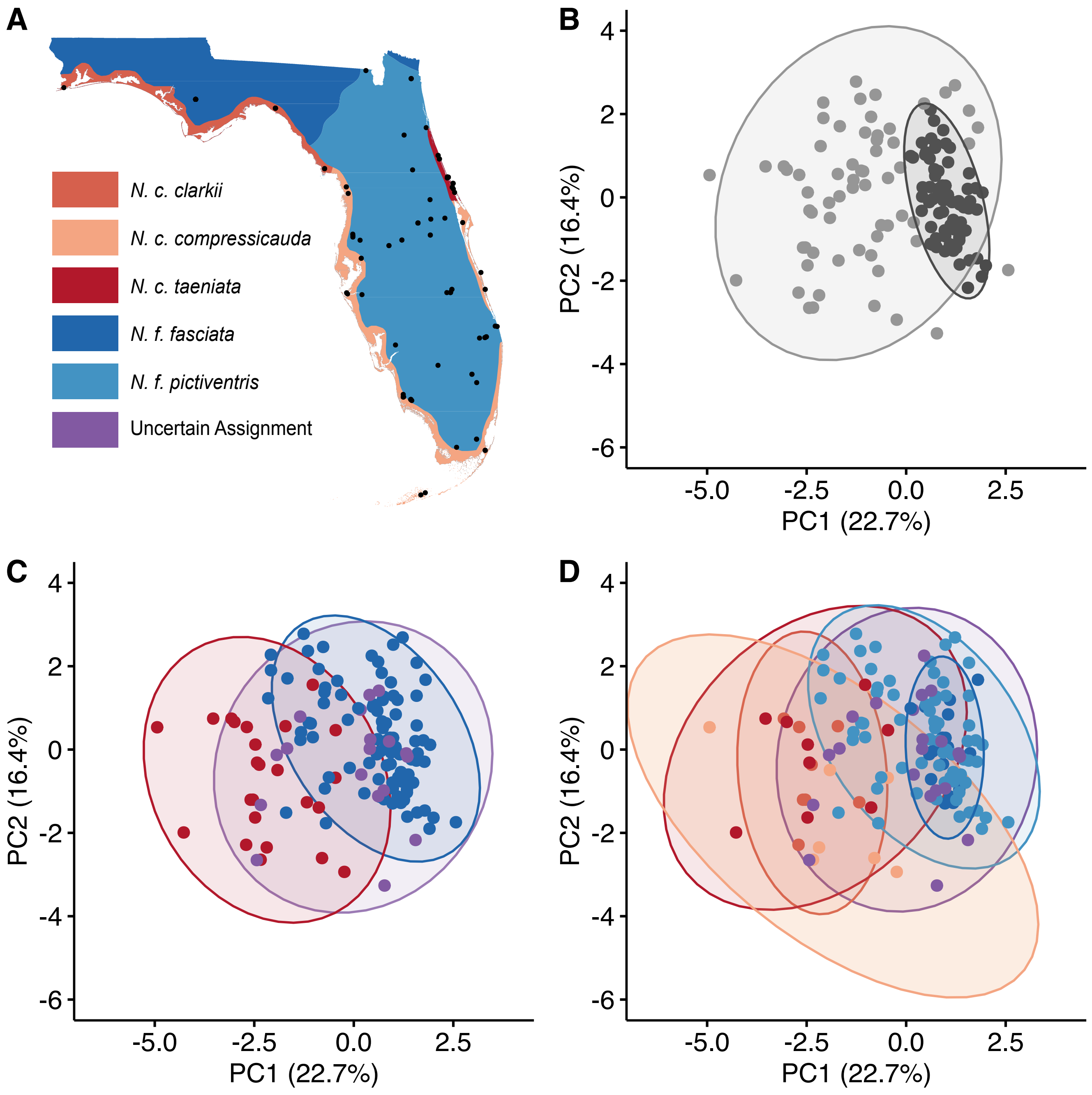

Fig S8. Morphology of the *N. fasciata-clarkii* complex. (A) Sampling map of individuals with morphological data. (B–D) PCA of morphological data with (B) mclust inferred clusters and (C) colored by identification upon capture for species and (D) subspecies.

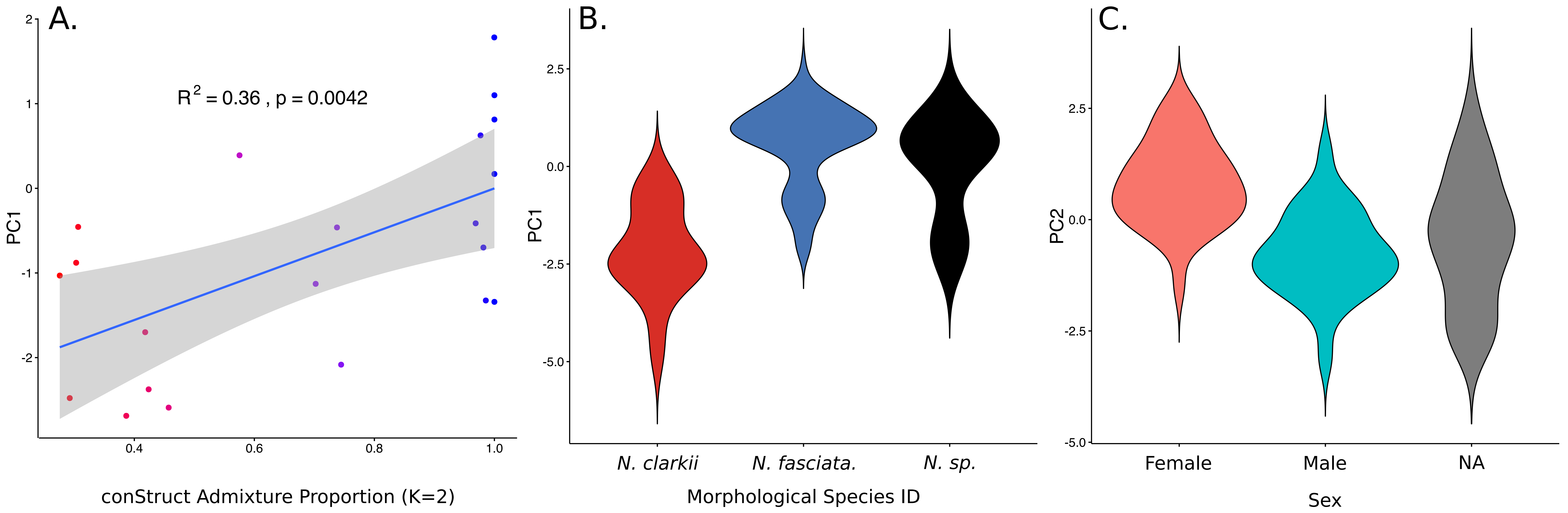

Fig S9. (A) Morphological PC1 regressed against conStruct admixture proportions. (B & C) Violin plots demonstrating divergence in (B) PC1 between species and (C) PC2 between sexes given model support by AIC.
